# Dietary Methionine Regulates Hepatic Autophagy and Apoptosis via m^6^A Methylation in Juvenile *Megalobrama amblycephala*

**DOI:** 10.3390/antiox14111327

**Published:** 2025-11-03

**Authors:** Qianwen Sun, Linjie Qian, Chuntao Xue, Qiushuang Ren, Wenqiang Jiang, Yan Lin, Siyue Lu, Zhengyan Gu, Linghong Miao

**Affiliations:** 1 Wuxi Fisheries College, Nanjing Agricultural University, Wuxi 214081, China; sqwww0824@163.com (Q.S.); qianlinjiejie@gmail.com (L.Q.); 17634364808@163.com (C.X.); m18315992812@163.com (Q.R.); 2 Key Laboratory of Freshwater Fisheries and Germplasm Resources Utilization, Ministry of Agriculture and Rural Affairs, Freshwater Fisheries Research Center, Chinese Academy of Fishery Sciences, Wuxi 214081, China; jiangwenqiang@ffrc.cn (W.J.); liny@ffrc.cn (Y.L.); lusiyue@ffrc.cn (S.L.); guzhengyan@ffrc.cn (Z.G.)

**Keywords:** *Megalobrama amblycephala*, all-plant-protein diet, methionine, m^6^A methylation, *wtap*/*fto*

## Abstract

This study investigated the epigenetic mechanisms through which graded levels of dietary methionine (Met) regulates growth, muscle quality, and health in juvenile *Megalobrama amblycephala* fed an all-plant-protein diet. Dietary Met supplementation improved growth performance in a dose-dependent manner and enhanced muscle nutritional quality, particularly protein content and amino acid composition. Optimal Met intake also strengthened hepatic antioxidant defenses, stabilized mitochondrial function, and modulated plasma metabolite profiles, including metabolites associated with antioxidant, anti-inflammatory, and antimicrobial activity. Epigenetic analysis revealed that dietary Met influenced hepatic N^6^-methyladenosine (m^6^A) RNA methylation and the expression of genes involved in autophagy and apoptosis, suggesting that these molecular pathways contribute to the observed physiological benefits. Collectively, these findings indicate that appropriate dietary Met of 10.1 g/kg not only supports growth and nutrient utilization but also promotes metabolic and cellular homeostasis through epigenetic regulation. This work provides novel insights into the nutritional and molecular strategies for improving the health and performance of juvenile *M. amblycephala* under plant-based feeding regimes, with potential implications for sustainable aquaculture practices.

## 1. Introduction

In recent years, the growing significant challenges to the sustainable development of aquaculture and the feed industry have led to an increasing interest in developing alternative non-grain protein sources (e.g., plant proteins, insects, fungi, microalgae, and single-cell proteins from bacteria) to replace fish meal [1]. Among these, plant proteins have become the dominant protein source in aquafeeds due to their wide availability and cost-effectiveness [2]. However, The application of plant proteins in aquafeeds is often limited by anti-nutritional factors and deficiencies in essential amino acids (EAA), which restrict their effectiveness as complete fish meal replacements [3,4]. Supplementing plant-based diets with these limiting amino acids, tailored to the species’ nutritional requirements, can substantially improve protein utilization [5,6,7].

Methionine (Met), a vital sulfur-containing EAA for aquatic species and the first limiting amino acid in plant protein-based diets, is crucial in promoting somatic growth and improving feed conversion efficiency. Beyond its fundamental function as a precursor for protein synthesis, Met is intricately involved in various metabolic processes, including immune regulation and antioxidant defense mechanisms [8,9,10]. Moreover, it is a primary methyl group donor, contributing significantly to biochemical reactions and epigenetic modifications. In aquatic animals, Met is metabolized through four main pathways: (1) it functions directly as a substrate for protein translation [10]; (2) it is converted into S-adenosylmet (SAM), a universal methyl donor involved in the methylation of proteins, DNA, and RNA, and can be remethylated to regenerate Met [11]; (3) upon activation to SAM, it contributes to polyamine biosynthesis, leading to the formation of spermidine and spermine [12]; (4) through transmethylation its intermediate product homocysteine (Hcy) is further metabolized to produce glutathione (GSH) and taurine, both of which show strong antioxidant properties [13]. These metabolic pathways underscore the key role of Met in protein synthesis, methyl donor (SAM), and antioxidant precursors (GSH/taurine) in aquatic animals.

N^6^-methyladenosine (m^6^A), the most abundant and evolutionarily conserved internal modification in eukaryotic mRNA, is a methylation at the nitrogen-6 position of adenosine and is dynamically regulated by methyltransferases (writers, e.g., methyltransferase like 3 (*mettl3*) and methyltransferase like 14 (*mettl14*)), demethylases (erasers, e.g., fat mass and obesity-associated protein (*fto*) and alkylation repair homolog 5 (*alkbh5*)), and m^6^A-binding proteins (readers, e.g., YTH domain family member (*ythdf1*/*2*/*3*) and embryonic lethal abnormal vision-like protein1 (*elavl1*)) [14,15]. This epitranscriptomic mark is regulated dynamically and reversibly by a specialized enzymatic machinery comprising m^6^A modification regulates various post-transcriptional processes, including pre-mRNA splicing, 3′-end processing, translational regulation, nuclear export, mRNA degradation, and the biogenesis of non-coding RNAs. Accordingly, m^6^A has regulated diverse physiological and pathological pathways, such as tumorigenesis, immune homeostasis, and metabolic disorders (obesity) [16]. Met, as an important methyl group donor, is converted to SAM and participates directly in regulating m^6^A methylation in vivo. In mammals, supplementation with lysine and Met in low-protein diets has been shown to modulate lipid metabolism and reduce fat deposition in lambs by downregulating the levels of methyltransferases (*mettl3*, *mettl14*) and demethylases (*fto*, *alkbh5*) [17]. Similarly, selenomethionine (SeMet) has been reported to alleviate hepatic oxidative stress and inflammation in laying hens by reducing *mettl3*-mediated m^6^A methylation of nuclear factor erythroid-2-related factor 2 (*nrf2*), thus enhancing liver antioxidant and anti-inflammatory capacity [18].

Blunt snout bream (*Megalobrama amblycephala*), commonly known as Wuchang bream, is a widely favored freshwater herbivorous fish species, with considerable potential and high economic value for aquaculture development due to its low production cost, rapid growth, high survival rate and high proportion of edible flesh. *M. amblycephala* is naturally distributed in the Yangtze River, China and is primarily cultured in freshwater polyculture systems across the country [19]. Existing literature indicates that species-specific characteristics, developmental stage, and rearing conditions influence the dietary Met requirement in fish. For example, juvenile *M. amblycephala* (3.34 ± 0.03 g) have been reported to require 8.4–8.5 g/kg Met when fed low-fishmeal diets containing 5% fishmeal [20], whereas fingerlings (101.80 ± 1.30 g) under similar dietary conditions show a lower requirement of 7.4–7.6 g/kg [21]. However, under the current demand for sustainable aquaculture, the Met requirement and its functional effects in juvenile *M. amblycephala* fed fishmeal-free, all-plant-protein diets remain unclear. Notably, no study has yet linked methionine supplementation to epigenetic (m^6^A) regulation in fish fed all-plant diets. Therefore, this study examined the optimal dietary Met demand for juvenile *M. amblycephala* under a completely plant-based diet and evaluated the potential epigenetic mechanisms by which Met (as a methyl donor) regulates nutrient metabolism and health in this species.

## 2. Materials and Methods

### 2.1. Ethics Approval and Consent to Participate

All experimental protocols, methods, and feeding procedures were approved by the Institutional Animal Care and Use Committee of the Freshwater Fisheries Research Center, Chinese Academy of Fishery Sciences. All animal handling and experimental procedures were conducted in accordance with the approved ethical guidelines.

### 2.2. Fish and Diets

This study evaluated the effects of graded levels of DL-Met supplementation in plant protein-based diets on juvenile *M. amblycephala* (Huahai No. 1 strain). Six isonitrogenous and isoenergetic diets were formulated with increasing DL-Met levels, resulting in measured dietary Met concentrations of 4.4 g/kg (CON), 7.0 g/kg (MET_1_), 8.6 g/kg (MET_2_), 10.1 g/kg (MET_3_), 12.5 g/kg (MET_4_), and 14.2 g/kg (MET_5_), respectively. The detailed composition of the basal diet and corresponding amino acid profiles are provided in Table A1 and Table 1.

### 2.3. Feeding Trial and Management

Approximately 480 healthy juvenile *M. amblycephala* with uniform body weight (initial mean weight: 12.34 ± 0.07 g) were randomly allocated into six experimental groups, each comprising four replicates. The feeding trial was conducted under a controlled indoor temperature-regulated recirculating aquaculture system at the Nanquan Facility of the Freshwater Fisheries Research Center, affiliated with the Chinese Academy of Fishery Sciences (Wuxi, China). After a one-week acclimatization phase in indoor glass tanks (water capacity: 300 L), the formal 10-week feeding trial was initiated. Fish were hand-fed to apparent satiation thrice daily (07:00, 12:00, and 17:00), allowing clear observation of the fish’s feeding behavior and activity through the transparent glass walls. When the fish ceased to respond actively to the feed, feeding was stopped to ensure that all pellets were completely consumed without residue. One-third of the tank water was replaced bi-daily to maintain optimal water conditions. The water quality during the feeding trial was explained in Appendix B.1.

### 2.4. Sample Collection

After 24 h of fasting, all fish were anesthetized using 3-aminobenzoic acid ethyl ester methanesulfonate (MS-222) at the concentration of 100 mg/L for sampling procedures. Growth performance and feed conversion ratio were recorded based on total biomass and fish count/tank. Blood samples were obtained from three fish per tank via caudal vein puncture using 1.0 mL syringes pre-treated with sterilized sodium heparin. Plasma and liver tissues were collected and processed for biochemical, metabolomic, and molecular analyses. Dorsal muscle samples were collected post skin removal (both flanks) for nutritional components and hydrolyzed amino acids determination. Moreover, three other fish were selected from each tank for immediate liver dissection, and mitochondrial membrane potential (MMP) and reactive oxygen species (ROS) levels were measured. The sample collection procedure was explained in Appendix B.2. Equal amounts of liver tissue from three fish originating from the same rearing glass tank were pooled, and four pooled liver samples from each treatment group were used for determining hepatic m^6^A levels, SAM contents and m^6^A methylation sequencing.

### 2.5. Laboratory Analysis

#### 2.5.1. Growth Performance Calculation

Final body weight (FBW, g) = W_t_/N;

Weight gain rate (WGR, %) = 100 × (W_t_ − W_0_)/W_0_;

Specific growth rate (SGR, % day^−1^) = 100 × (ln W_t_ − ln W_0_)/t;

Feed conversion ratio (FCR) = F/(W_t_ − W_0_).

Note: W_0_ is the total weight of each tank of fish at the beginning of the culture test (g); W_t_ is the total weight of each tank of fish at the end of the feeding trial (g); t is the feeding days (d); F is the total intake of feed (dry basis, g); N is the number of fish per tank at the end of the culture test.

#### 2.5.2. Assessment of Plasma Biochemical and Hepatic Antioxidant Indexes

Plasma concentrations of alanine aminotransferase (ALT), aspartate aminotransferase (AST), lactate dehydrogenase (LDH), and glucose (GLU) were quantified using a fully automated biochemical analyzer (Mindray Bio-Medical Electronics Co., Ltd., Shenzhen, China) in combination with commercially available assay kits (Shanghai Zhicheng Biotechnology Co., Ltd., Shanghai, China). The supernatants of 10% liver homogenates were collected for determining enzymatic activity of catalase (CAT) and levels of glutathione (GSH), malondialdehyde (MDA), and total antioxidant capacity (T-AOC) via specific commercial kits (Nanjing Jiancheng Bioengineering Institute, Nanjing, China).

#### 2.5.3. Determination of Hepatic MMP and ROS Production

Mitochondria were isolated, and the MMP and intracellular ROS levels were quantified using the JC-1 assay and the DCFH-DA method using commercially available kits, respectively, by the supplied instructions (Shanghai Biyuntian Biotechnology Co., Ltd., Shanghai, China). Fluorescence microscopy (Leica Microsystems, Wetzlar, Germany) was employed for visualization. Intracellular ROS oxidizes the non-fluorescent DCFH to its fluorescent form (DCF), indicating oxidative stress. For MMP analysis, high membrane potential leads to JC-1 aggregation within the mitochondrial matrix, emitting red fluorescence. However, reduced membrane potential prevents JC-1 aggregation, resulting in green fluorescence due to monomeric forms. Fluorescence intensity was quantified via Image J software (v1.8.0), and results were expressed accordingly.

#### 2.5.4. Determination of Muscle Nutritional Components

Muscle nutrient components including dry matter content, crude protein content, crude fat content, and ash content were determined with reference to the methods specified in the national standard of the atmospheric pressure drying method, Kjeldahl nitrogen fixation, Soxhlet extraction, and the calcination method of 560 °C.

#### 2.5.5. Determination of Hydrolyzed Amino Acids (HAA) in Muscle and FAA in Plasma

The amino acid composition in muscle (including taurine) were quantified after acid hydrolysis using an amino acid analyzer of Agilent 1100 (Agilent, Santa Clara, CA, USA). High-performance liquid chromatography (HPLC) was employed to measure FAA composition and content in plasma using a Waters 2695 HPLC system (Waters, Milford, MA, USA). The sample treatment procedure was explained in Appendix B.3.

#### 2.5.6. LC-MS-Based Untargeted Metabolomics Analysis of Plasma

Plasma metabolites were analyzed using LC-MS. using a Vanquish UHPLC system coupled with a mass spectrometer (Q Exactive™ HF-X, ThermoFisher Scientific, Waltham, MA, USA). The plasma metabolomics analysis procedure was explained in Appendix B.4.

#### 2.5.7. Analysis of Hepatic m^6^A Level and SAM Content

The EpiQuik™ m^6^A RNA Methylation Quantification Kit (Colorimetric; Beijing Chunye Technology Co., Ltd., Beijing, China) was used to quantify N6-methyladenosine (m^6^A) modifications in hepatic RNA. Liver SAM content was quantified using a fish SAM ELISA kit (Shanghai ChaoRui Biotechnology Co., Ltd., Shanghai, China). Absorbance was measured at 450 nm using a microplate reader, and SAM concentrations were calculated accordingly. The hepatic m^6^A level and SAM content analysis procedures were explained in Appendix B.5.

#### 2.5.8. M^6^A Methylation Sequencing

Trizol (Invitrogen, CA, USA) was used to extract and purify RNA from liver tissue. The Epicentre Ribo-Zero Gold Kit (Illumina, San Diego, CA, USA) was used to remove the rRNA of total RNA that met the conditions, and then the RNA was fragmented using the magnesium ion interruption Kit (NEBNext^®^ Magnesium RNA Fragmentation Module, Ipswich, MA, USA) under high-temperature conditions. Finally, m^6^A-seq and RNA-seq libraries were constructed and sequenced according to the reported method [22].

#### 2.5.9. Hepatic Gene Expression Analysis

Gene expression levels in liver tissue were determined via qRT-PCR analysis [23]. Target gene transcripts were quantified using a CFX96 Touch real-time PCR detection system (Bio-Rad, Hercules, CA, USA). Primers were designed using the NCBI Primer Blast tool (Table A2) and synthesised by Shanghai BioEngineering Co., Ltd (Shanghai, China). *β-actin* was selected as the housekeeping gene, and relative transcription levels were analyzed using the 2^−ΔΔCT^ method.

### 2.6. Statistical Analyses

Data was statistically analyzed via SPSS 27.0 (SPSS Inc., Chicago, IL, USA). Experimental data were examined for normality using the Shapiro–Wilk test, confirming adherence to the assumptions of normal distribution and homogeneity of variance. Substantial variations among groups were analyzed using one-way analysis of variance (ANOVA) followed by Duncan’s multiple comparison test, with a significance threshold set at *p* < 0.05. For comparison between the CON and MET_3_ groups, an independent samples *t*-test was conducted. A *p*-value < 0.05 was depicted as statistically significant, while *p* < 0.01 was interpreted as highly significant. General Linear Model (Univariate) was conducted to estimate effect size (the Partial Eta Squared, η^2^). Data are shown as mean ± SEM.

## 3. Results

### 3.1. Growth Performance and Feed Efficiency in Response to Dietary Met Supplementation

**Figure 1:**
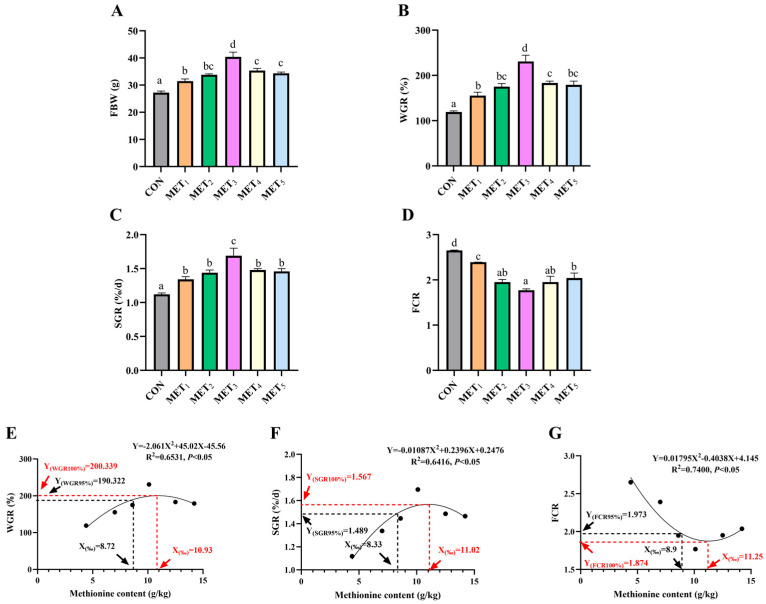
Effects of methionine supplementation in all-plant-protein diet on growth performance and feed utilization of juvenile *M. amblycephala*. (**A**) final body weight; (**B**) weight gain rate; (**C**) specific growth rate; (**D**) feed conversion ratio; (**E**–**G**) quadratic regression fitting curve of optimal methionine content in all-plant-protein diet (WGR, SGR, FCR). The various superscripts represent remarkable differences across six groups (*p* < 0.05). Effect size classification based on Partial Eta Squared (η^2^): Small effect: 0.01 ≤ η^2^ < 0.06; Medium effect: 0.06 ≤ η^2^ < 0.14; Strong effect: η^2^ ≥ 0.14.

Dietary Met supplementation induced quadratic changes in growth performance, with FBW, WGR, and SGR increasing and then decreasing, while FCR exhibited the opposite trend. All Met-supplemented groups showed significantly higher FBW than CON, with MET_3_ achieving the highest value (*p* < 0.05, η^2^ = 0.866). Similarly, WGR (η^2^ = 0.855) and SGR (η^2^ = 0.766) were elevated in all Met groups, peaking in MET_3_ (*p* < 0.05). FCR decreased significantly in all Met groups compared with CON, with MET_3_ showing the lowest value (*p* < 0.05, η^2^ = 0.837, Figure 1A–D). Quadratic regression analysis (Figure 1E–G) revealed that the optimal dietary Met inclusion level was approximately 11 g/kg. Specifically, the levels for maximizing weight gain rate (WGR) and specific growth rate (SGR) and minimizing feed conversion ratio (FCR) were 10.93 g/kg (corresponding to a maximum WGR of 200.34%), 11.02 g/kg (maximum SGR of 1.567%/d), and 11.25 g/kg (minimum FCR of 1.874), respectively.

### 3.2. Plasma Biochemical Indicators and Hepatic Antioxidant Profile Against Dietary Met Supplementation

**Figure 2:**
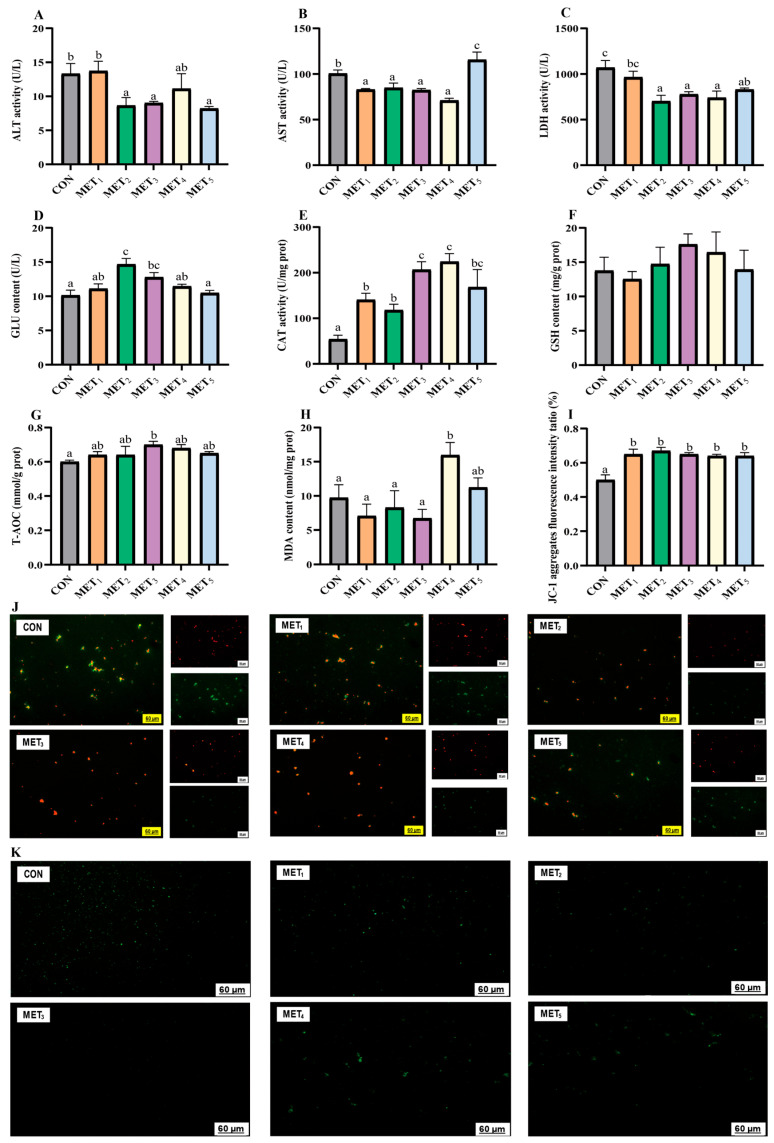
Effects of methionine supplementation in all-plant-protein diet on plasma biochemical index and hepatic reactive oxygen species of juvenile *M. amblycephala*. (**A**) alanine aminotransferase; (**B**) aspartate aminotransferase; (**C**) lactate dehydrogenase; (**D**) glucose; (**E**) catalase; (**F**) glutathione; (**G**) total antioxidant capacity; (**H**) malondialdehyde; (**I**,**J**) hepatic mitochondrial membrane potential ((**I**) ratio of red fluorescence intensity to red and green fluorescence intensity; (**J**) the red fluorescence is JC-1 aggregates, the green fluorescence is JC-1 monomer;) (**K**) hepatic reactive oxygen species in mitochondria. The various superscripts represent remarkable differences across six groups (*p* < 0.05).

Progressive addition of Met in the all-plant-protein diet was associated with a general decline in plasma ALT and LDH activities. ALT levels were remarkably reduced in the MET_2_, MET_3_, and MET_5_ groups compared to the CON and MET_1_ groups (*p* < 0.05, η^2^ = 0.311, Figure 2A). Similarly, LDH activities in MET_2_ through MET_5_ were substantially lower than those in the CON group, while MET_2_, MET_3_, and MET_4_ showed lower values than MET_1_ (*p* < 0.05, η^2^ = 0.442, Figure 2C). AST activity depicted a biphasic pattern, initially decreasing and increasing with elevated Met supplementation. AST levels in the MET_1_-_4_ groups were statistically distinct from those in the CON and MET_5_ groups, with the MET_5_ group showing elevated levels relative to the CON group (*p* < 0.05, η^2^ = 0.624, Figure 2B). Plasma GLU concentrations showed a transient elevation followed by a decline as Met levels increased. The MET_2_ group represented the highest GLU value, which was significantly higher than those in the CON, MET_1_, MET_4_, and MET_5_ groups. The MET_3_ group also displayed higher GLU levels than both the CON and MET_5_ groups (*p* < 0.05, η^2^ = 0.442, Figure 2D).

Liver antioxidant capacity was modulated by dietary Met levels, showing a biphasic response. Both CAT activity and T-AOC increased with moderate Met supplementation and declined at higher levels. Both MET_3_ and MET_4_ groups showed the highest CAT activity, showing significant variations relative to the CON, MET_1_, and MET_2_ groups, while the MET_5_ group also depicted significantly elevated CAT activity relative to the CON group (*p* < 0.05, η^2^ = 0.482, Figure 2E). T-AOC was maximized in the MET_3_ group, which presented considerably higher values than the CON group (*p* < 0.05; η^2^ = 0.171, Figure 2G). MDA levels in the MET_4_ group were remarkably elevated in comparison to CON and MET_1-3_ groups (*p* < 0.05, η^2^ = 0.272, Figure 2H), indicating increased lipid peroxidation. No significant differences were observed in hepatic GSH levels among the experimental groups (*p* > 0.05, η^2^ = 0.067, Figure 2F), suggesting that GSH content remained stable despite variations in Met intake. The MMP levels were positively correlated with the red fluorescence/red-to-green fluorescence intensity ratio (Figure 2I,J), and compared to the CON group, the MMP levels in the Met addition groups were considerably higher (*p* < 0.05). The ROS levels in the liver were positively correlated with the green fluorescence intensity, as shown in Figure 2K. With increasing Met addition, the fluorescence intensity of ROS initially decreased and then increased.

### 3.3. Changes in Muscle Nutritional Components and HAA Profiles in Response to Dietary Met Supplementation

**Figure 3:**
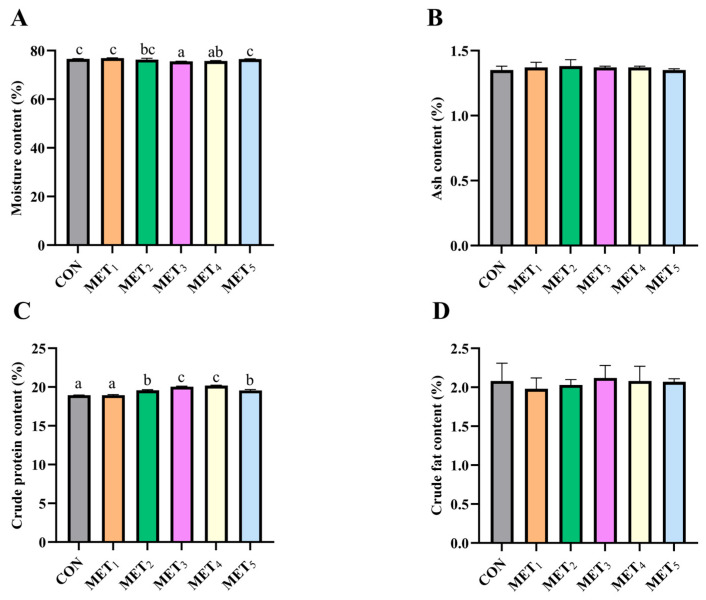
Effects of methionine supplementation in all-plant-protein diet on muscle nutritional components of juvenile *M. amblycephala*. (**A**) moisture content in muscle; (**B**) crude ash content in muscle; (**C**) crude protein content in muscle; (**D**) crude fat content in muscle. The various superscripts represent remarkable differences across six groups (*p* < 0.05).

Variations in dietary Met levels exerted a differential influence on muscle composition in juvenile *M. amblycephala*. As Met concentration increased, a non-linear trend was observed in moisture and crude protein content. Muscle moisture content showed an initial decline followed by an increase. Both MET_3_ and MET_4_ groups showed lower moisture levels than the CON, MET_1_, and MET_5_ groups, with the MET_3_ group also showing a significant reduction relative to MET_2_ group (*p* < 0.05; Figure 3A). Crude protein content increased at moderate Met levels, initially rising and then declining with higher Met inclusion, with the MET_3_ and MET_4_ groups exhibiting the highest values (*p* < 0.05; Figure 3C).

As presented in Table 2, the hydrolyzed Met content in muscle increased with rising dietary Met levels and reached a stable phase. The MET_2_–_5_ groups showed higher Met levels compared to the CON group (*p* < 0.05). Among the non-essential amino acids (NEAA), hydrolyzed serine, taurine, and cysteine concentrations initially increased and decreased with higher dietary Met levels. Serine levels were elevated in the MET_3_–_5_ groups relative to the CON and MET_1_ groups (*p* < 0.05). Taurine concentration reached its highest level in the MET_4_ group and differed from the values observed in the CON, MET_1_, MET_2_, and MET_5_ groups (*p* < 0.05). Taurine levels in the MET_3_ and MET_5_ groups were also higher than that in the CON, MET_1_, and MET_2_ groups (*p* < 0.05). Cysteine content was higher in the MET_4_ group and exceeded the levels in the CON and MET_1_ groups (*p* < 0.05).

### 3.4. Changes in Plasma FAA Profiles in Response to Dietary Met Supplementation

As presented in Table 3, no differences were observed in plasma free Met levels among the groups. However, the levels of histidine, threonine, and arginine were affected by dietary Met content. Dietary Met supplementation induced distinct alterations in the plasma concentrations of FAAs in juvenile *M. amblycephala*. A progressive increase in plasma taurine levels was observed with increasing Met levels, with the MET_4_ and MET_5_ groups showing significantly elevated concentrations relative to the CON and MET_1_ groups (*p* < 0.05). Plasma serine levels demonstrated a biphasic trend, increasing initially and declining. The MET_2_ and MET_3_ groups displayed the highest serine concentrations, which were statistically greater than those in the CON, MET_1_, and MET_5_ groups (*p* < 0.05), while the MET_4_ group also showed elevated levels relative to CON and MET_1_ (*p* < 0.05).

In addition, the levels of plasma free histidine and free threonine were increased across all Met-supplemented groups (*p* < 0.05), while the arginine level in the MET_3_ group was significantly higher than in MET_4_ and MET_5_ (*p* < 0.05). Glycine content was significantly higher in the MET_1_ group than in the CON, MET_2_, and MET_3_ groups (*p* < 0.05). Aspartic acid concentration was significantly reduced in the MET_5_ group compared to all other treatments (*p* < 0.05). In case of glutamic acid, plasma concentrations were significantly elevated in the MET_3_ and MET_4_ groups in comparison to other groups (*p* < 0.05). The total FAA levels in all Met-treated groups (MET_1–5_) were significantly elevated in contrast to the CON group (*p* < 0.05). A comparable trend was observed for plasma EAA, where MET_1–4_ groups demonstrated increased concentrations relative to the CON and MET_5_ groups (*p* < 0.05).

### 3.5. Plasma Metabolomic Analysis

**Figure 4:**
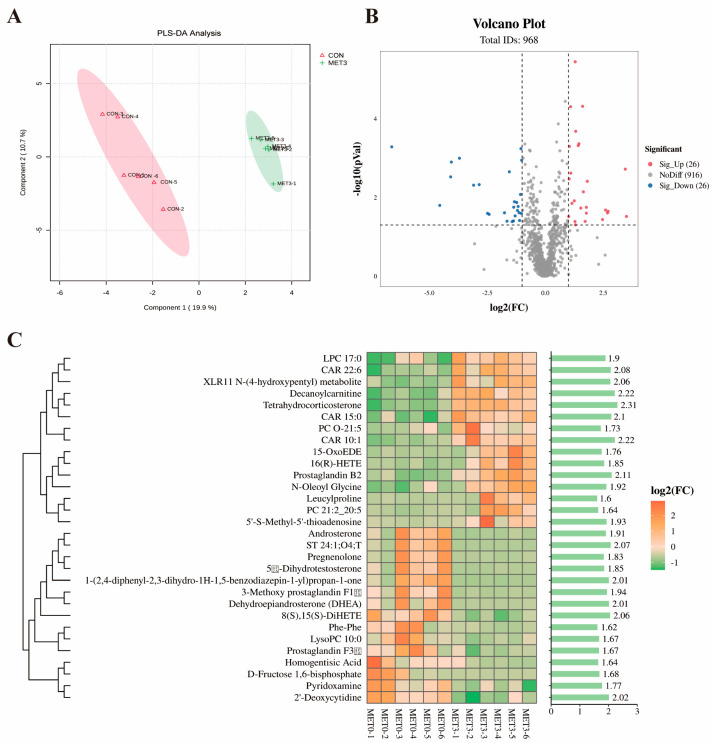
Plasma metabolomic analysis between CON and MET_3_ groups. (**A**) partial least squares discriminant (PLS-DA) analysis: pink represents CON group and green represents MET3 group; (**B**) volcanic map: red indicates significant increase, blue indicates significant decrease, and gray indicates no significant difference; (**C**) differential metabolite heatmap and VIP bar graphs: the higher the VIP value, the greater the contribution of metabolites to sample classification.

Figure 4A shows that partial least squares discriminant analysis (PLS-DA) revealed distinct clustering and separation of plasma metabolites between the CON and MET_3_ groups, indicating clear metabolic differences. As shown in the volcano plot (Figure 4B), approximately 52 differential metabolites were detected in the MET_3_ group relative to the CON group, with 26 metabolites showing increased and 26 revealing decreased levels.

Based on the absolute value of Log_2_(MET_3_/CON), the Heatmap analysis of differential metabolites identified 30 differential metabolites between the MET_3_ and CON groups, including the top 14 metabolites with significantly increased levels and the top 16 metabolites with significantly decreased levels. As shown in Figure 4C, the metabolites with significantly increased levels in the MET_3_ group’s plasma included tetrahydrocorticosterone, 16(R)-hydroxyeicosatetraenoic acid (16(R)-HETE), prostaglandin B2, leucylproline, and 5′-S-methyl-5′-thioadenosine (MTA) (*p* < 0.05), while the metabolites with significantly decreased levels included androsterone, dehydroepiandrosterone (DHEA), lysophosphatidylcholine (LysoPC), prostaglandin F3β, and homogentisic acid (*p* < 0.05).

### 3.6. Hepatic m^6^A Methylation Analysis

**Figure 5:**
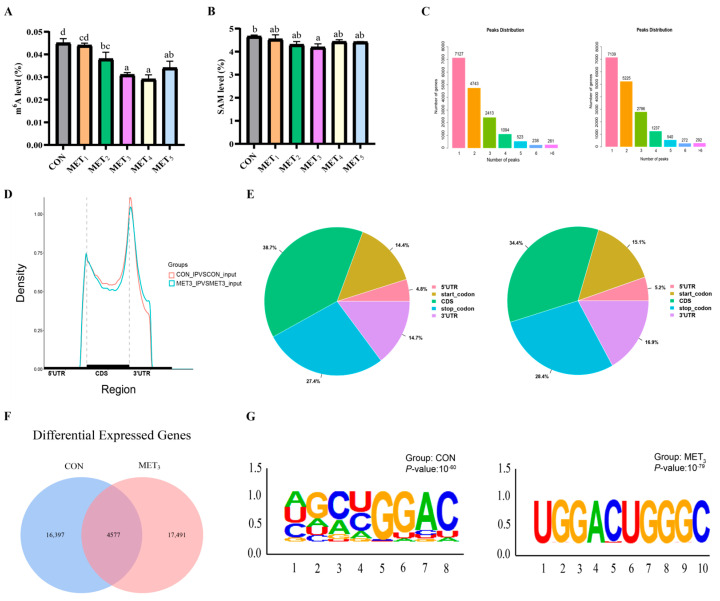
Hepatic m^6^A methylation analysis between CON and MET_3_ groups. For MeRIP-Seq, m^6^A peaks were identified with exomePeak2 (v1.12.0; *p*  <  1 × 10^−5^) and annotated by ANNOVAR (Ver. 20240617). Significant peaks met |log_2_FC|  >  1 and FDR  <  0.05. Motifs were discovered with HOMER (v4.11) and visualized via ggseqlogo/ggplot2. Differential peaks were analyzed with DiffBind (v2.8.0). For RNA-Seq, transcripts were assembled with StringTie (v1.3.4), quantified with RSEM (v1.2.19), and DEGs identified with DESeq2 (v1.20.0; |log_2_FC| ≥ 2, FDR  <  0.05). (**A**) m^6^A level, the various superscripts represent remarkable differences across six groups (*p* < 0.05); (**B**) S-adenosyl methionine content, the various superscripts represent remarkable differences across six groups (*p* < 0.05); (**C**) the number of m^6^A modification sites of CON (left) and MET_3_ (right) group genes; (**D**) differences in the cumulative distribution of m^6^A peaks in the CON and MET_3_ groups; (**E**) distribution position of m^6^A peaks in CON (left) and MET_3_ (right) groups; (**F**) Venn diagram of m^6^A modification sites in mRNAs of CON and MET_3_ groups; (**G**) analysis and comparison of m^6^A motif enrichment in mRNAs of CON and MET_3_ groups.

An upward adjustment in dietary Met levels was associated with a non-linear pattern in hepatic m^6^A methylation and SAM content in juvenile *M. amblycephala*, characterized by an initial decline followed by an elevation. The m^6^A levels in the MET_3_ and MET_4_ groups were lower than those in the CON, MET_1_, and MET_2_ groups, while the MET_5_ group showed reduced levels relative to the CON and MET_1_ groups. Furthermore, the MET_2_ group revealed decreased m^6^A levels in comparison to the CON group (*p* < 0.05; Figure 5A). Hepatic SAM content in the MET_3_ group was also lower than that in the CON group (*p* < 0.05; Figure 5B). The gene-wide distribution of m^6^A peaks in the CON group revealed that 43% of genes harbored a single m^6^A modification site, and 87% possessed between one and three sites. A comparable distribution pattern was identified in the MET_3_ group, where 40% of genes contained a single site and 86% contained one to three sites (Figure 5C). Enrichment analysis of m^6^A peak locations demonstrated a preferential accumulation at the 3′ UTR initiation sites and the 5′ UTR terminal sites. The coding sequence (CDS) regions displayed a relatively lower peak density. Compared to the CON group, a reduction in m^6^A peak density at the 3′ UTR start region was observed in the MET_3_ group (Figure 5D). The proportional distribution of m^6^A methylation sites in the liver of the CON group was as follows: 38.7% in the CDS region, 27.4% in the terminator region, 14.4% in the start codon region, 14.7% in the 3′ UTR, and 4.8% in the 5′ UTR. In the MET_3_ group, the proportions were 34.4%, 28.4%, 15.1%, 16.9%, and 5.2%, respectively (Figure 5E). Approximately 20,974 and 22,068 m^6^A methylation sites were identified in the CON and MET_3_ groups, respectively, with 4577 sites shared between both groups, as illustrated in Figure 5F. Motif analysis revealed the presence of the common m^6^A motif GGAC in both the CON and MET_3_ groups, consistent with the typical m^6^A modification pattern (Figure 5G).

### 3.7. Combined Analysis of m^6^A-Seq and RNA-Seq

**Figure 6:**
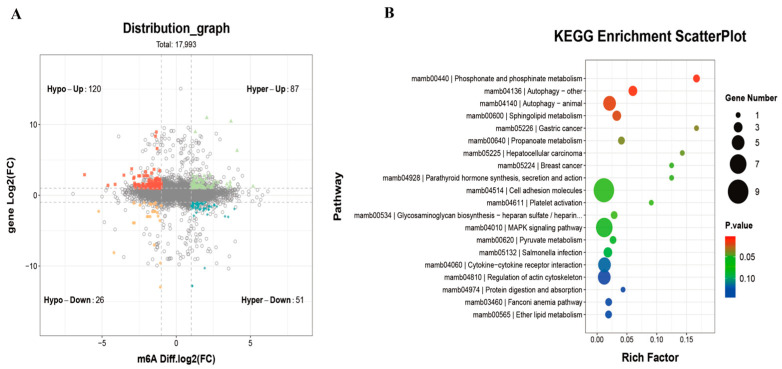
Association analysis between differential m^6^A methylation modified genes and differential RNA transcription and expression genes. Functional enrichment of peak-related genes and DEGs was performed using GO.db (v3.14.0) and KEGG (Release 101; adjusted *p*  <  0.05). Differential expression and m^6^A peaks were further analyzed with edgeR (v4.4.1; |log_2_FC|  >  1, *p*  <  0.05). (**A**) four quadrant diagram of association analysis between differential m^6^A methylation modified genes and differential RNA transcription and expression genes; (**B**) KEGG richness analysis of differential expressing genes by joint m^6^A-seq and RNA-seq analysis.

A combined analysis of differentially modified m^6^A methylation genes and differentially expressed mRNAs between the CON and MET_3_ groups revealed 284 genes with similar changes in both m^6^A methylation and transcriptional expression (Figure 6A). These genes were classified into four regulatory categories: a subset of 87 genes, including *casp3b*, *foxo4*, and *atg9a*, showed upregulation of m^6^A methylation and mRNA expression (Hyper-up); 51 genes, such as *cep112*, *apba1b*, and *eva1ba*, showed increased m^6^A methylation along with reduced mRNA expression (Hyper-down); 120 genes, including *csdc2b*, *marchf2*, and *bach1b*, demonstrated decreased m^6^A methylation alongside elevated mRNA expression (Hypo-up); and 26 genes, such as *mapta*, *cadpsb*, and *cracdlb*, displayed downregulation of both m^6^A methylation and mRNA expression (Hypo-down). KEGG pathway enrichment analysis (Figure 6B) indicated that these overlapping genes were primarily enriched in pathways related to autophagy (Autophagy-Other; Autophagy-Animal), sphingolipid metabolism, hepatocellular carcinoma, and MAPK signaling.

### 3.8. Expression of Regulatory Genes Related to Liver Methylation and Inflammatory Injury

**Figure 7:**
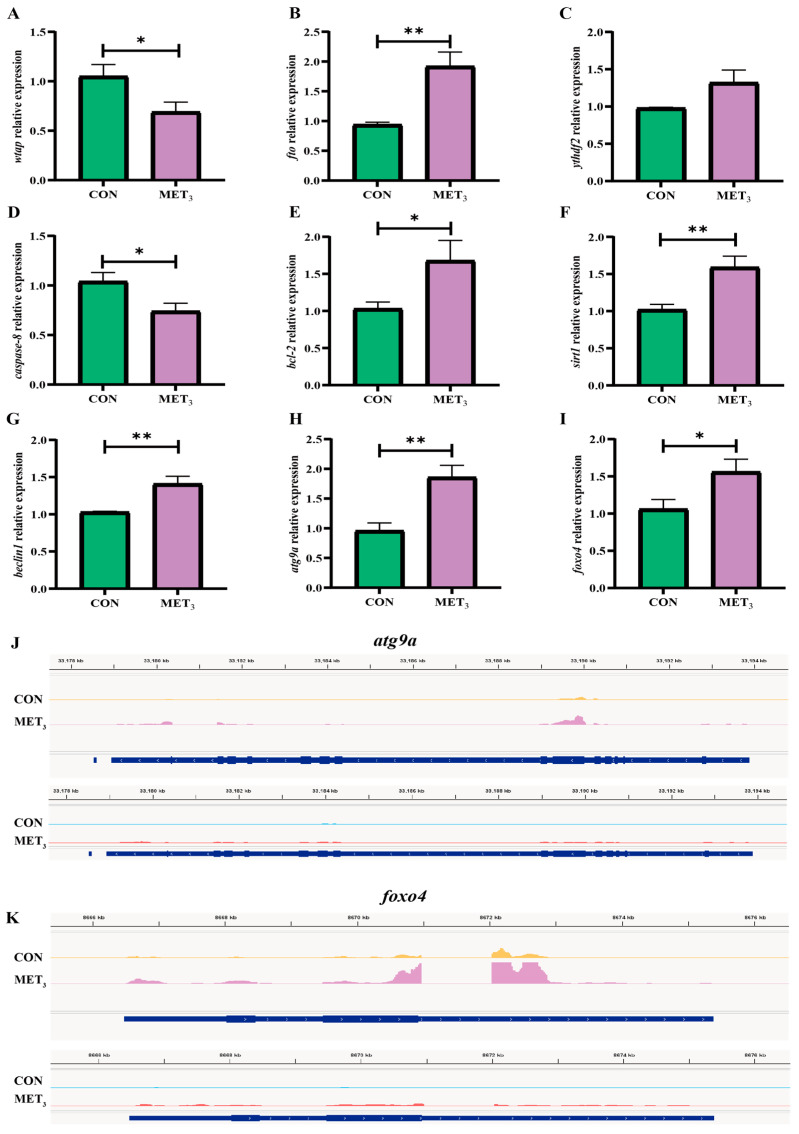
Expression of regulatory genes related to liver methylation and inflammatory injury. (**A**) wilms tumor1-associating protein; (**B**) fat mass and obesity-associated protein; (**C**) YTH domain family member2; (**D**) cysteine aspartate-specific protease 8; (**E**) B-cell lymphoma-2; (**F**) silent information regulator 1; (**G**) benzyl chloride 1; (**H**) autophagy-related protein 9a; (**I**) forkhead box protein O4; (**J**,**K**) peak abundance of m^6^A (IP, yellow and peak) and expression (Input, blue and red) in *atg9a* and *foxo4* in liver of CON and MET_3_ groups. * means the significant difference between the two groups (Independent *t*-test, *p*  <  0.05), and ** means the extremely significant difference between the two groups (Independent *t*-test, *p*  <  0.01).

Compared to the CON group, the expression of the methyltransferase *watp* in the liver of juveniles in the MET_3_ group was significantly downregulated (*p* < 0.05, Figure 7A), while the expression of the demethylase *fto* was upregulated (*p* < 0.01, Figure 7B). There were no substantial variations in the expression of *ythdf2* (*p* > 0.05, Figure 7C). In comparison to the CON group, the expression of *caspase-8* in the liver of juveniles in the MET_3_ group was significantly downregulated (*p* < 0.05, Figure 7D). However, the expressions of *bcl-2* and *foxo4* were upregulated (*p* < 0.05, Figure 7E,I), and the expressions of *sirt1*, *beclin1*, and *atg9a* were extremely upregulated (*p* < 0.01, Figure 7F–H). IGV visualization analysis showed the m^6^A peak expression patterns of *atg9a* and *foxo4.* High methylation occurred in the 3′ UTR region of *atg9a* and *foxo4*, resulting in changes in gene expression. The expression of *atg9a* and *foxo4* in the MET_3_ group was substantially higher than in the CON group (Figure 7J,K).

## 4. Discussion

### 4.1. Effect of Different Met Levels in Plant-Based Diets on Growth Performance and Antioxidant Capacity

Amino acids are essential for protein synthesis and play a critical role in the growth and development of aquatic species. Appropriate dietary methionine (Met) enhances growth in juvenile *M. amblycephala* under low fishmeal diets, whereas excessive supplementation may inhibit growth [24], a pattern also observed in grouper (*Epinephelus coioides*) [25], grass carp (*Ctenopharyngodon Idella*) [26], and black sea bream (*Sparus macrocephalus*) [27]. Consistent with these findings, Met supplementation in plant-based diets improved growth and reduced feed conversion ratio in this study. Previous studies reported that juvenile *M. amblycephala* of two sizes groups (3.34 ± 0.03 g and 101.80 ± 1.30 g) require 8.4–8.5 g/kg Met or 7.4–7.6 g/kg in low fishmeal diets [20,21]. Based on secondary regression analysis, the estimated dietary Met requirement for achieving optimal WGR, SGR, and minimum FCR in juvenile *M. amblycephala* fed all-plant-protein diets was approximately 11 g/kg. These values were higher than typical levels observed in fishmeal-based diets, reflecting the relatively low Met content of plant-derived protein sources. Plasma ALT, AST, and LDH are important indicators of liver health. Higher levels of ALT, AST, and LDH suggest that the liver may be experiencing oxidative stress, leading to functional impairment [23]. Studies have reported that Met can alleviate the increase in ALT and AST levels in the plasma of ricefield eel (*Monopterus albus*) caused by feeding low-protein diets and improve liver damage induced by low-protein diets [28]. The present study suggested that appropriate Met supplementation (8.6 g/kg and 10.1 g/kg) enhanced hepatic function and improved antioxidant capacity in fish fed plant-based protein diets, as indicated by reduced plasma ALT, AST, and LDH levels.

ROS continuously accumulate in mitochondria under oxidative stress, leading to cell damage [29]. They are primarily generated by mitochondrial complex I and III, with the production sites affected by MMP [30]. MMP plays an essential role in cellular homeostasis as it drives the ATP production in organelles [31]. Aberrant MMP, whether elevated or reduced, has been associated with mitochondrial dysfunction, promoting cellular apoptosis and necrosis [32]. Met restriction has been demonstrated to induce pronounced mitochondrial impairment in human osteosarcoma cells, characterized by a clear reduction in MMP and elevation in ROS generation [33]. In this study, Met supplementation increased the red fluorescence and red-to-green fluorescence ratio in the mitochondrial matrix of juvenile *M. amblycephala* livers, indicating that MMP became more stable. Similarly, fluorescence detection of ROS in the liver showed that Met supplementation in plant-based diets reduced the liver’s ROS content, especially when the Met level was 10.1 g/kg, which alleviated oxidative stress damage and maintained MMP stability in juvenile *M. amblycephala*. The findings were consistent with hepatic antioxidant enzyme analysis, indicating that dietary Met supplementation in plant-based formulations enhanced liver antioxidant enzyme activities in juvenile *M. amblycephala*. Importantly, 10.1 g/kg supplementation levels were associated with elevated CAT activity and T-AOC. Similarly, Met supplementation has been reported to reduce hepatic oxidative stress in large yellow croaker (*Larimichthys crocea*) subjected to high-fat diets by decreasing MDA concentrations and enhancing T-AOC, thus highlighting hepatic antioxidant defenses [34]. These findings revealed that methionine enhances antioxidant capacity by influencing the SAM/SAH levels and regulating the expressions and activities of antioxidant genes. Simultaneously, elevated methionine promotes the trans-sulfuration pathway, increasing cysteine and glutathione (GSH) levels. Together, these mechanisms link methionine nutrition to epigenetic regulation, mitochondrial performance, and redox homeostasis. Furthermore, this study observed that when the Met content in plant-based diets was high (≥12.5 g/kg), a large amount of MDA was generated in the liver. This phenomenon has been reported in both mammals and aquatic animals, where excessive Met causes damage to rats, increasing ROS content in liver mitochondria and damaging mitochondrial DNA [35]; excessive Met in yellow catfish (*Pelteobagrus fulvidraco*) diets also induces cell apoptosis [36].

### 4.2. Effects of Different Met Levels in Plant-Based Protein Diets on Muscle Nutrient Composition and Blood Metabolite Profiles of Juvenile M. amblycephala

Met functions as the initiating amino acid during protein translation and serves as a rate-limiting factor in protein biosynthesis [37]. Adequate Met supplementation in plant-based protein diets has been shown to enhance muscle crude protein content in species such as hybrid crucian carp (*Carassius auratus gibelio*) and Nile tilapia (*Oreochromis niloticus*) [5,38]. In this study, it was similarly found that when the Met content in plant-based protein diets ranged from 8.6 g/kg to 14.2 g/kg, the protein content in the muscles of juvenile *M. amblycephala* substantially increased, suggesting that Met is involved in promoting muscle protein synthesis in juvenile *M. amblycephala*. Further examination of muscle amino acid composition demonstrated a progressive elevation in Met, taurine, cysteine, and serine concentrations with increasing dietary Met levels. Comparable trends have been reported in other teleost species; for instance, dietary Met supplementation elevated the intramuscular concentrations of free Met, cysteine, and serine in *C. idella* [26]. In studies of *C. auratus gibelio*, supplementing Met in low-fishmeal diets improved cysteine deposition in juvenile fish [39]. Furthermore, dietary Met concentration significantly influenced taurine accumulation in *C. carpiovar* muscle, showing a positive correlation with increased Met levels [11]. This effect may be attributed to the metabolic interconnection among sulfur-containing amino acids, as both taurine and cysteine are involved in the metabolic pathways of Met within the organism. Moreover, Met generates homocysteine through transmethylation, and in the homocysteine metabolic pathway, serine, through remethylation, provides methyl groups for Met synthesis and condenses with homocysteine to form cystathionine [40].

The FAA content in plasma is an important indicator reflecting the protein status of the animal body, as well as amino acid metabolism and consumption [9]. In the present study, elevated dietary Met levels facilitated the accumulation of free taurine in plasma. This observation suggests that the supplemented Met may have undergone metabolic conversion to taurine via the trans-sulfuration pathway in juvenile *M. amblycephala*. Similarly, supplementing Met in the plant-based protein diet of *O. niloticus* also increased the plasma taurine content, although the change was not statistically significant [41]. The Met level affects the content of most FAAs in plasma in the diet [42]. In this study, supplementing Met in the plant-based protein diet also caused changes in the plasma levels of histidine, threonine, and other amino acids in juveniles. This may be related to the excess or disproportionate intake of Met, which affects the utilization of other amino acids [43,44]. Moreover, this study found that appropriate Met supplementation could increase the total content of FAA, EAA, and NEAA in the plasma of juvenile *M. amblycephala*. However, the plasma Met content did not increase with the higher level of Met in the diet. Considering the observed elevation in hydrolyzed Met levels in muscle tissue, it is suggested that Met metabolism varies between blood and muscle. Met is primarily used for protein synthesis within muscle tissue and is metabolized into taurine through the trans-sulfuration pathway in the bloodstream and muscle.

Metabolites are small molecular compounds produced or consumed through metabolic processes in organisms, playing vital roles in energy supply, metabolic regulation, immune regulation, and other functions. 16(R)-HETE is a metabolite of the arachidonic acid metabolic pathway cytochrome P450 (CYP450), involved in blood pressure regulation, lipid synthesis, and inflammatory responses [45]. Studies have found that 16 (R)-HETE can inhibit CYP1A2 activity and reduce cardiac toxicity [46]. Prostaglandin B2, a bioactive lipid mediator derived from arachidonic acid, inhibits the accumulation of cyclic AMP in hepatocytes stimulated by glucagon and has anti-inflammatory effects [47]. Tetrahydrocortisol is a corticosteroid closely related to glucose and lipid metabolism, with anti-inflammatory and immune-regulatory effects [48]. Previous studies have shown that tetrahydrocortisol can alleviate acute skin inflammation in mice [49]. In the present study, plasma concentrations of 16(R)-HETE, prostaglandin B2, and tetrahydrocortisol were substantially elevated in the MET_3_ group of juvenile *M. amblycephala* relative to the CON group. These findings suggest that Met supplementation may enhance anti-inflammatory responses and immunological function in juvenile *M. amblycephala* consuming a plant-based protein diet, thus contributing to improved growth performance. Furthermore, an evident increase in plasma levels of MTA was observed in the MET_3_ group, along with an evident reduction in LysoPC concentrations. MTA is a metabolite produced through the polyamine pathway from SAM and can regulate cell proliferation, differentiation, and apoptosis processes [50]. MTA is also a protein methyltransferase inhibitor, regulating the transcription and activation of relevant genes by inhibiting H3K4 methylation [51]. LysoPC is a lipid mediator with regulatory functions in epigenetics, including regulating cell apoptosis and signal transduction. Studies have shown that LysoPC can induce cysteine protease-dependent cell death in mouse liver cells and trigger endoplasmic reticulum stress [52]. Therefore, it is predicted that supplementing an appropriate amount of Met in the plant-based protein diet improves resistance to oxidative damage in juvenile *M. amblycephala* by modulating MTA and LysoPC metabolism and involvement in the methylation pathway.

### 4.3. Met Regulates Hepatic Autophagy and Apoptosis in Juvenile M. amblycephala by Affecting Liver m^6^A Methylation Levels

M^6^A methylation is a key regulatory mechanism in RNA biology, influencing protein synthesis and enabling rapid modulation of dynamic cellular processes [53]. Liver is the primary organ responsible for Met metabolism, and evidence indicates that up to 85% of m^6^A methylation events and approximately 48% of Met metabolism occur in hepatic tissue [54]. The abundance of m^6^A reflects the overall methylation status within the examined tissue. SAM, a central metabolite in hepatic Met metabolism, is a crucial methyl donor in the m^6^A modification pathway [55]. This study found that Met decreased the total m^6^A levels and SAM content in the liver of juvenile *M. amblycephala*, which was different from the findings on Salmon alevins. These differences may result from variations in methionine requirements and physiological functions between marine and freshwater fish, or from the different types of methionine supplements (L-Met vs. DL-Met) used in the diets [56,57]. Met mainly participates in four physiological metabolic processes: protein synthesis, methylation, polyamine production, and trans-sulfuration. Combined with the increased muscle protein and plasma taurine content in juvenile *M. amblycephala*, it is speculated that with appropriate Met supplementation, its primary physiological function may involve protein metabolism and maintaining tissue health homeostasis, decreasing overall liver methylation.

This study further revealed that the m^6^A peak in the liver of the MET_3_ group was remarkably reduced in the CDS region and the promoter of the 3′ UTR, suggesting that the demethylation of Met likely occurs in the central region of the gene’s 3′ UTR. This could improve mRNA stability and translation efficiency or alleviate miRNA-mediated suppression of physiological function regulation. Therefore, Met possibly exerts regulatory effects by performing more refined and selective ‘demethylation’ of key genes. Methyltransferases catalyze the methylation activity of m^6^A modifications, while demethylases catalyze the demethylation of RNA substrates with m^6^A modifications [18]. Research shows that *wtap* can promote endoplasmic reticulum stress and apoptosis in cardiomyocytes by downregulating the m^6^A modification of activating transcription factor4 (*atf4*) mRNA, causing myocardial damage [58]. In hepatic ischemia–reperfusion injury (IRI), *fto* expression is significantly downregulated; however, upregulation of *fto* has been shown to reduce liver IRI by attenuating apoptosis and autophagy [59]. In this study, Met considerably reduced the m^6^A methylation levels in the liver of juvenile *M. amblycephala*, along with a significant decrease in *wtap* and an increase in *fto*, indicating a synergistic interaction between *wtap* and *fto* in the health impairment of juvenile *M. amblycephala*. Similar studies have also found that [Cu(ttpy-tpp)Br_2_]Br reduces SAM and S-adenosyl homocysteine levels in liver cells by inhibiting the Met cycle, thus inducing the senescence of cancer cells in the liver [60].

Autophagy represents a highly conserved catabolic mechanism in eukaryotic cells, responsible for the selective degradation and recycling of damaged intracellular components. This process is pivotal in maintaining cellular energy homeostasis and facilitating adaptive responses under conditions of nutritional deprivation [61]. Sirtuins (Sirts), a class of nicotinamide adenine dinucleotide (NAD^+^)-dependent deacetylases, are crucially involved in the regulation of cell propagation, programmed cell death, and autophagic activity [39]. *Sirt1* is the most studied and widely distributed member of the Sirt family and is closely related to autophagy, and the Foxo pathway also involves key physiological processes such as autophagy, differentiation, and oxidative stress. In cancer research, it has been found that *foxo4* can inhibit cancer cell proliferation, regulate the cell cycle, and promote autophagy and apoptosis [62]. The Atg ubiquitin-like conjugation system is involved in the autophagy initiation, extension, and maturation stages. *Atg9a*, an essential integral membrane protein, functions as a central regulatory node in autophagosome membrane biogenesis and is crucially involved in mediating membrane remodeling required for autophagosome closure, thus facilitating the terminal stages of autophagy [63]. Moreover, *beclin1* is a specific gene involved in autophagy, which positively regulates the occurrence of autophagy [64]. There is a close relationship between genes regulating autophagy. *Sirt1* can upregulate *foxo4* expression by regulating the Foxo pathway, thus improving the organism’s ability to resist oxidative stress [65]. *Sirt1* induces BCL2 interacting protein 3 (*bnip3*) expression by deacetylating *foxo3*, thus triggering autophagy by promoting the dissociation of the anti-apoptotic gene *bcl-2* and the *beclin1* complex [66]. The Foxo pathway can also upregulate the activation of Atg genes through transcription.

Cellular autophagy and apoptosis are basic regulatory mechanisms by which organisms respond to internal and external stressors. Apoptosis, a tightly controlled form of programmed cell death, is used to preserve cellular homeostasis and eliminate damaged or unwanted cells in a regulated manner. *Caspase-8*, a member of the cysteine protease family, is considered an apoptosis promoter. Once activated, it can induce cell apoptosis [67]. Evidence indicates that autophagy may be initiated as a cellular adaptive response preceding apoptosis, particularly under growth factor deprivation or exposure to cytotoxic agents, to reduce cellular damage and delay the onset of programmed cell death [68]. Supplementing with Met can reduce the apoptosis signal of *aip56* in the head kidney leukocytes of European bass, showing lower cysteine aspartate-specific protease 3 (*caspase-3*) activity [69]. Curcumin, when used as a methyl donor in cardiomyocytes of diabetic cardiomyopathy mice, has been demonstrated to induce autophagy through activation of the AMPK signaling pathway. This activation facilitates the dissociation of the *bcl-2-beclin1* complex, thus attenuating apoptotic processes [70]. In the present study, dietary Met supplementation elicited activation of key autophagy-related *sirt1*, *atg9a*, and *foxo4* transcription factors in juvenile *M. amblycephala* maintained on a plant-based diet. This intervention was associated with an elevated expression of the *bcl-2* and a reduction in *caspase-8* expression. These results indicate that Met may suppress apoptosis by enhancing autophagic mechanisms, thus improving the growth impairment of juveniles caused by feeding a plant-based diet.

## 5. Conclusions

**Figure 8:**
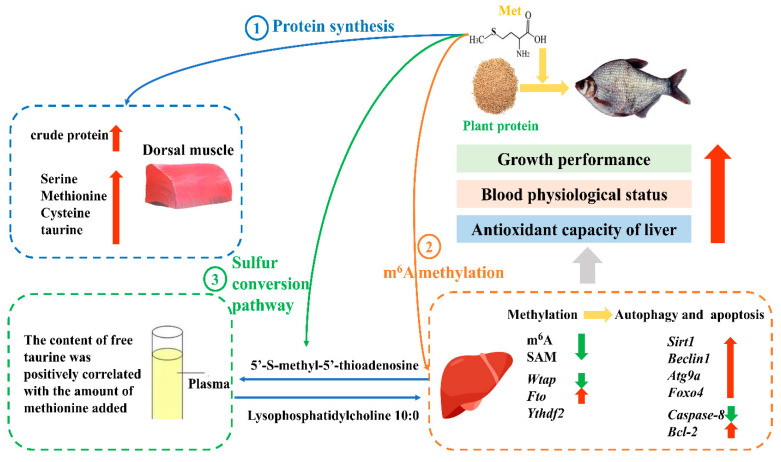
The epigenetic mechanisms of dietary methionine regulating growth and health in juvenile *Megalobrama amblycephala.* The red arrows represent the significant increase in indicators, while the green arrows represent the significant decrease in indicators.

This study concluded that an optimal dietary Met level of 10.1 g/kg enhances growth rate, improves hematological parameters, and strengthens hepatic antioxidant potential in juvenile *M. amblycephala*. This supplementation modulates the profiles of muscle hydrolyzed amino acids and plasma free amino acids, elevates plasma metabolites associated with antioxidant, anti-inflammatory, and antibacterial activities, and promotes hepatic autophagy through the *wtap*/*fto* demethylation pathway. These effects collectively contribute to reduced hepatocellular apoptosis and improved overall fish health (Figure 8). This study proposes a potential regulatory mechanism linking methionine, m^6^A methylation, and autophagy/apoptosis. However, functional validation experiments (e.g., gene knockdown or inhibitor treatments) were not conducted to substantiate the causal relationships. Future investigations should incorporate functional assays to validate the roles of key regulatory factors and to further clarify the causal pathways through which methionine modulates m^6^A methylation and the associated autophagy and apoptosis processes.

## Figures and Tables

**Table 1:** Amino acid composition of experimental feed.

g/100 g	CON	MET_1_	MET_2_	MET_3_	MET_4_	MET_5_
* Methionine	0.44	0.70	0.86	1.01	1.25	1.42
* Histidine	1.00	1.00	1.07	1.06	1.07	1.02
* Threonine	1.24	1.25	1.28	1.25	1.26	1.24
* Valine	1.94	2.00	1.98	1.90	1.98	1.99
* Phenylalanine	2.04	2.12	2.08	2.04	2.05	2.06
* Arginine	3.84	4.02	3.95	3.91	3.91	3.93
* Isoleucine	1.48	1.54	1.54	1.50	1.51	1.53
* Leucine	2.53	2.63	2.60	2.54	2.57	2.58
* Lysine	2.39	2.48	2.48	2.39	2.49	2.42
Aspartic acid	4.21	4.44	4.42	4.28	4.30	4.37
Glutamic acid	8.42	8.81	8.69	8.46	8.55	8.58
Serine	1.54	1.58	1.56	1.56	1.56	1.52
Glycine	1.68	1.71	1.73	1.69	1.72	1.70
Alanine	1.60	1.67	1.66	1.62	1.64	1.63
Taurine	0.05	0.05	0.07	0.07	0.07	0.06
Tyrosine	1.04	1.06	1.03	1.01	1.02	1.00
Cysteine	0.17	0.16	0.14	0.15	0.15	0.15
Proline	1.93	2.02	1.97	1.80	1.75	1.90

Note: The data in the table were measured average values (*n* = 3). * essential amino acids. dietary CON, an experimental diet with the DL-Met concentrations of 4.4 g/kg; MET_1_, an experimental diet with the DL-Met concentrations of 7.0 g/kg; MET_2_, an experimental diet with the DL-Met concentrations of 8.6 g/kg; MET_3_, an experimental diet with the DL-Met concentrations of 10.1 g/kg; MET_4_, an experimental diet with the DL-Met concentrations of 12.5 g/kg; MET_5_, an experimental diet with the DL-Met concentrations of 14.2 g/kg.

**Table 2:** Content of hydrolyzed amino acids in muscle of juvenile *M. amblycephala* (wet weight).

g/100 g	CON	MET_1_	MET_2_	MET_3_	MET_4_	MET_5_	η^2^
Essential amino acids							
Methionine	0.20 ± 0.10 ^a^	0.34 ± 0.00 ^ab^	0.45 ± 0.01 ^b^	0.41 ± 0.00 ^b^	0.44 ± 0.06 ^b^	0.43 ± 0.07 ^b^	0.549
Histidine	0.66 ± 0.01	0.66 ± 0.01	0.65 ± 0.01	0.65 ± 0.01	0.66 ± 0.02	0.65 ± 0.01	0.189
Threonine	0.76 ± 0.02	0.76 ± 0.01	0.76 ± 0.02	0.77 ± 0.01	0.77 ± 0.01	0.77 ± 0.02	0.071
Valine	1.02 ± 0.00	1.02 ± 0.01	0.99 ± 0.04	0.97 ± 0.01	1.00 ± 0.01	0.99 ± 0.02	0.296
Phenylalanine	0.86 ± 0.02	0.88 ± 0.01	0.86 ± 0.02	0.88 ± 0.01	0.88 ± 0.02	0.88 ± 0.02	0.180
Arginine	1.16 ± 0.06	1.17 ± 0.03	1.15 ± 0.01	1.13 ± 0.01	1.15 ± 0.04	1.19 ± 0.03	0.140
Isoleucine	0.90 ± 0.03	0.91 ± 0.01	0.91 ± 0.02	0.92 ± 0.01	0.92 ± 0.02	0.91 ± 0.02	0.060
Leucine	1.51 ± 0.04	1.55 ± 0.03	1.53 ± 0.04	1.55 ± 0.02	1.56 ± 0.03	1.55 ± 0.03	0.141
Lysine	1.72 ± 0.06	1.77 ± 0.02	1.74 ± 0.05	1.80 ± 0.03	1.79 ± 0.04	1.80 ± 0.05	0.191
Nonessential amino acids							
Aspartic acid	2.25 ± 0.06	2.29 ± 0.04	2.26 ± 0.04	2.34 ± 0.01	2.28 ± 0.05	2.28 ± 0.06	0.161
Glutamic acid	3.12 ± 0.11	3.16 ± 0.06	3.13 ± 0.08	3.18 ± 0.01	3.20 ± 0.08	3.18 ± 0.09	0.071
Serine	0.65 ± 0.02 ^a^	0.66 ± 0.01 ^a^	0.68 ± 0.01 ^ab^	0.70 ± 0.01 ^b^	0.71 ± 0.01 ^b^	0.70 ± 0.01 ^b^	0.625
Glycine	0.95 ± 0.03	0.94 ± 0.03	0.97 ± 0.01	0.95 ± 0.03	0.91 ± 0.03	1.01 ± 0.05	0.301
Alanine	1.15 ± 0.04	1.15 ± 0.03	1.15 ± 0.01	1.15 ± 0.01	1.16 ± 0.02	1.18 ± 0.02	0.067
Taurine	0.30 ± 0.01 ^a^	0.30 ± 0.01 ^a^	0.31 ± 0.00 ^a^	0.36 ± 0.01 ^bc^	0.37 ± 0.01 ^c^	0.35 ± 0.01 ^b^	0.876
Tyrosine	0.47 ± 0.04	0.49 ± 0.02	0.62 ± 0.01	0.60 ± 0.03	0.62 ± 0.04	0.61 ± 0.03	0.680
Cysteine	0.04 ± 0.00 ^a^	0.05 ± 0.00 ^a^	0.06 ± 0.01 ^ab^	0.05 ± 0.01 ^ab^	0.07 ± 0.01 ^b^	0.06 ± 0.01 ^ab^	0.531
Proline	0.61 ± 0.03	0.62 ± 0.02	0.65 ± 0.02	0.61 ± 0.02	0.64 ± 0.02	0.67 ± 0.02	0.365
Total amino acids	18.34 ± 0.66	18.74 ± 0.26	18.87 ± 0.35	19.02 ± 0.13	19.14 ± 0.43	19.22 ± 0.45	0.200
Total essential amino acids	7.63 ± 0.27	7.90 ± 0.09	7.89 ± 0.20	7.95 ± 0.10	8.03 ± 0.18	7.97 ± 0.22	0.147
Total nonessential amino acids	10.70 ± 0.40	10.84 ± 0.18	10.98 ± 0.15	11.06 ± 0.03	11.12 ± 0.26	11.25 ± 0.25	0.270

Note: the various superscripts represent remarkable differences across six groups (*p* < 0.05). η^2^, Partial Eta Squared. A higher partial eta-squared indicates a larger magnitude of difference or effect, and the commonly cited thresholds for small, medium, and large effects are approximately 0.01, 0.06, and 0.14.

**Table 3:** Content of free amino acids in plasma of juvenile *M. amblycephala*.

g/L	CON	MET_1_	MET_2_	MET_3_	MET_4_	MET_5_	η^2^
Essential amino acids							
Methionine	21.00 ± 2.00	21.33 ± 2.40	20.33 ± 2.02	18.00 ± 1.73	20.00 ± 2.00	15.00 ± 1.53	0.382
Histidine	89.67 ± 4.10 ^a^	111.67 ± 0.67 ^b^	115.00 ± 0.00 ^b^	113.00 ± 6.35 ^b^	118.33 ± 1.45 ^b^	97.00 ± 6.93 ^a^	0.751
Threonine	25.50 ± 0.29 ^a^	36.00 ± 2.31 ^c^	29.67 ± 0.33 ^b^	30.67 ± 0.88 ^b^	31.00 ± 0.00 ^b^	32.67 ± 0.88 ^bc^	0.809
Valine	35.67 ± 2.73	33.67 ± 1.20	36.33 ± 2.60	31.33 ± 0.88	33.00 ± 0.58	32.67 ± 0.67	0.342
Phenylalanine	19.00 ± 0.58	21.33 ± 1.33	21.50 ± 0.86	19.33 ± 0.33	21.00 ± 0.00	20.00 ± 0.58	0.463
Arginine	52.67 ± 1.76 ^ab^	50.67 ± 3.18 ^ab^	52.67 ± 4.41 ^ab^	57.67 ± 3.18 ^b^	42.00 ± 2.08 ^a^	44.33 ± 3.93 ^a^	0.575
Isoleucine	18.00 ± 0.58	17.33 ± 0.33	18.67 ± 0.88	17.67 ± 0.33	18.33 ± 0.67	19.33 ± 0.67	0.368
Leucine	32.67 ± 1.20	32.33 ± 0.88	36.33 ± 2.91	31.67 ± 1.33	37.00 ± 2.08	35.00 ± 0.58	0.425
Lysine	40.33 ± 0.33	42.00 ± 1.53	43.33 ± 0.33	42.33 ± 0.33	37.67 ± 0.88	38.00 ± 4.00	0.417
Nonessential amino acids							
Aspartic acid	24.67 ± 1.20 ^b^	24.00 ± 1.53 ^b^	21.00 ± 0.58 ^b^	23.67 ± 0.88 ^b^	24.00 ± 0.58 ^b^	17.00 ± 1.53 ^a^	0.740
Glutamic acid	52.67 ± 0.88 ^c^	47.67 ± 1.76 ^bc^	26.67 ± 3.18 ^a^	63.00 ± 0.58 ^d^	62.50 ± 0.87 ^d^	41.50 ± 5.48 ^b^	0.913
Serine	3.67 ± 0.88 ^a^	5.33 ± 1.86 ^a^	13.67 ± 1.45 ^c^	16.33 ± 2.73 ^c^	11.33 ± 0.67 ^bc^	7.00 ± 0.58 ^ab^	0.811
Glycine	17.66 ± 0.88 ^ab^	21.33 ± 0.33 ^c^	16.33 ± 0.3 ^a^	16.33 ± 1.86 ^a^	18.33 ± 0.33 ^abc^	20.00 ± 1.15 ^bc^	0.632
Alanine	43.00 ± 2.65	46.00 ± 1.00	41.67 ± 2.40	38.67 ± 2.03	38.67 ± 1.33	44.33 ± 1.45	0.507
Taurine	156.33 ± 2.02 ^a^	165.00 ± 6.93 ^a^	168.67 ± 11.35 ^ab^	174.33 ± 1.20 ^ab^	189.00 ± 7.94 ^b^	190.00 ± 3.00 ^b^	0.640
Tyrosine	30.00 ± 2.65	34.00 ± 0.58	33.67 ± 2.33	31.67 ± 1.86	34.00 ± 1.73	31.33 ± 1.20	0.255
Cysteine	4.00 ± 1.00	4.67 ± 1.76	2.67 ± 1.76	4.67 ± 0.33	4.67 ± 1.33	4.33 ± 0.33	0.142
Proline	9.00 ± 0.58	13.00 ± 3.78	12.00 ± 2.52	11.33 ± 1.45	11.33 ± 0.33	12.00 ± 1.00	0.157
Total amino acids	682.17 ± 2.89 ^a^	737.00 ± 9.29 ^bc^	720.67 ± 10.74 ^b^	753.00 ± 5.03 ^c^	762.17 ± 11.97 ^c^	709.83 ± 8.92 ^b^	0.673
Total essential amino acids	290.50 ± 5.48 ^a^	325.33 ± 3.84 ^b^	331.67 ± 8.41 ^b^	315.33 ± 4.26 ^b^	326.33 ± 5.24 ^b^	298.00 ± 5.00 ^a^	0.835
Total nonessential amino acids	391.67 ± 2.60 ^a^	411.67 ± 6.89 ^ab^	389.00 ± 9.87 ^a^	437.67 ± 4.33 ^b^	435.83 ± 8.66 ^b^	411.82 ± 13.18 ^ab^	0.610

Note: The various superscripts represent remarkable differences across six groups (*p* < 0.05). η^2^, Partial Eta Squared. A higher partial eta-squared indicates a larger magnitude of difference or effect, and the commonly cited thresholds for small, medium, and large effects are approximately 0.01, 0.06, and 0.14.

## Data Availability

The authors confirm that the data supporting the findings of this study are available within the manuscript and table.

## Abbreviations

The following abbreviations are used in this manuscript:

16(R)-HETE	16(R)-hydroxyeicosatetraenoic acid
8(S),15(S)-DiHETE	8(S),15(S)-dihydroxyeicosatetraenoic acid
Alkbh5	Alkylation repair homolog 5
ALT	Alanine aminotransferase
AST	Aspartate aminotransferase
Atf4	Activating transcription factor4
Atg9a	Autophagy-related protein 9a
Bcl-2	B-cell lymphoma-2
Beclin1	Benzyl chloride 1
Bnip3	BCL2interacting protein3
Caspase-3	Cysteine aspartate-specific protease 3
Caspase-8	Cysteine aspartate-specific protease 8
CAT	Catalase
CDS	Coding sequence
CYP	Arachidonic acid metabolic pathway cytochrome P450
DHEA	Dehydroepiandrosterone
EAA	Essential amino acid
Elavl1	Embryonic lethal abnormal vision-like protein1
FAA	Free amino acid
FBW	Final body weight
FCR	Feed conversion ratio
Foxo3	forkhead box protein O 3
Foxo4	forkhead box protein O 4
Fto	Fat mass and obesity-associated protein
GLU	Glucose
GSH	Glutathione
HAA	Hydrolyzed amino acid
HPLC	High-performance liquid chromatography
IRI	Ischemia–reperfusion injury
LDH	Lactate dehydrogenase
LysoPC	Lysophosphatidylcholine
m^6^A	N-methyladenosine
MDA	Malondialdehyde
Met	Methionine
Mettl14	Methyltransferase like 14
Mettl3	Methyltransferase like 3
MMP	Mitochondrial membrane potential
MTA	5′-S-methyl-5′-thioadenosine
NAD^+^	Nicotinamide adenine dinucleotide
NEAA	Non-essential amino acids
Nrf2	Nuclear factor erythroid-2-related factor2
PLS-DA	Partial least squares discriminant analysis
ROS	Reactive oxygen species
SAM	S-adenosylmet
SeMet	Selenomethionine
SGR	Specific growth rate
Sirt1	Sirtuins silent information regulator 1
T-AOC	Total antioxidant capacity
WGR	Weight gain rate
Wtap	Wilms tumor1-associating protein
Ythdf	YTH domain family member

## Appendix A

**Table A1:** Experimental feed formulation and nutritional composition (%, dry basis).

Ingredient	CON	MET_1_	MET_2_	MET_3_	MET_4_	MET_5_
Soybean meal ^1^	20.00	20.00	20.00	20.00	20.00	20.00
Rapeseed meal ^1^	20.00	20.00	20.00	20.00	20.00	20.00
Cottonseed meal ^1^	19.00	19.00	19.00	19.00	19.00	19.00
Cottonseed protein concentrate	15.00	15.00	15.00	15.00	15.00	15.00
Wheat flour ^1^	14.00	13.75	13.50	13.25	13.00	12.75
Rice bran ^1^	1.80	1.80	1.80	1.80	1.80	1.80
Soybean oil	6.50	6.50	6.50	6.50	6.50	6.50
Calcium dihydrogen phosphate	1.00	1.00	1.00	1.00	1.00	1.00
Mineral premix ^2^	1.00	1.00	1.00	1.00	1.00	1.00
Choline chloride	0.30	0.30	0.30	0.30	0.30	0.30
Bentonite	0.20	0.20	0.20	0.20	0.20	0.20
DMPT	0.50	0.50	0.50	0.50	0.50	0.50
lysine	0.70	0.70	0.70	0.70	0.70	0.70
Methionine	0.00	0.25	0.50	0.75	1.00	1.25
Total	100.00	100.00	100.00	100.00	100.00	100.00
Nutritional ingredient						
Crude fat	8.62	8.43	8.88	8.60	8.56	8.89
Crude protein	36.43	36.36	36.95	36.36	36.39	36.92
Gross energy	17.06	17.02	16.97	16.93	16.88	16.84

Note: ^1^ Provided by Beijing Dabeinong Technology Group Co., Ltd. (Huaian, China). Soybean meal: crude protein 46.7%, crude fat 4.25%; Rapeseed meal: crude protein 43.9%, crude fat 6.14%; Cottonseed meal: crude protein 55.6%, crude fat 4.49%; Wheat flour: crude protein 17.4%, crude fat 3.95%; Rice bran: crude protein 16.3%, crude fat 21.71%. ^2^ Provided by Beijing Dabeinong Technology Group Co., Ltd. (Huaian, China). Mineral (g/kg of premix) premix: calcium biphosphate, 20 g; sodium chloride, 2.6 g; potassium chloride, 5 g; magnesium sulfate, 2 g; ferrous sulfate, 0.9 g; zinc sulfate, 0.06 g; cupric sulfate, 0.02 g; manganese sulfate, 0.03 g; sodium selenate, 0.02 g; cobalt chloride, 0.05 g; potassium iodide, 0.004 g.

**Table A2:** Primer sequences used for qRT-PCR.

Gene Name	Primer Sequence (5′-3′)	L	E (%)	NCBI Number
*wtap*	F: AGAGCTCAAGAGCAGCCAAG R: GTTCAGAGGCCGTTGAAGGA	200	108.60	XM_048206845.1
*fto*	F: GATTCTGCAGCTGGTGGACT R: GTCTGTCTGTGCTGCTGTCT	181	103.50	XM_048184627.1
*alkbh5*	F: GATCGATGAGGTGGTTGCCA R: TACGTGTAGCCCTCTCCGAA	105	109.20	XM_048197746.1
*ythdf2*	F: GGACAAGTGGAAGGGACGTT R: TCCAGTGGAACCTCCTGAGT	141	100.40	XM_048191909.1
*caspase-8*	F: GCAGATGGACAAGGGAACG R: TGGCGAAAGGACTGGTAATG	137	109.80	XM_048200598.1
*bcl-2*	F: CGTCTACCTGGACAACCACA R: GCGTTTCTGTGCAATGAGTG	190	106.50	XM_048179299.1
*sirt1*	F: TCGGTTCATTCAGCAGCACA R: ATGATGATCTGCCACAGCGT	120	102.90	XM_048203988.1
*beclin1*	F: ATGAAAGCACGATGGAGGGTT R: CCCTCGCTGCTATCCAACTG	187	109.40	XM_048187618.1
*foxo4*	F: GCTTCACAGGGATCCACTCC R: AGCTTGGCTGCTGGACATAG	282	109.40	XM_048175885.1
*atg9a*	F: TCTACCTGTGCGCTTTCGTT R: CGGTTCCCTCCACGTTTGTA	156	108.80	XM_048200701.1
*β-actin*	F: TCGTCCACCGCAAATGCTTCTA R: CCGTCACCTTCACCGTTCCAGT	152	106.60	AY170122.2

Note: F: forward primer; R: reverse primer; L: amplification sequence length; E: amplification efficiency

## Appendix B

### Appendix B.1. Supplementary Explanation on Water Quality Index of the Feeding Trial

One-third of the tank water was replaced bi-daily to maintain optimal water conditions during the feeding trial. Water quality was monitored weekly, with the following parameters maintained: 26–28 °C temperature, dissolved O_2_ concentration ≥ 6 mg/L, ammonia nitrogen levels ≤ 0.05 mg/L, pH between 7.0 and 7.5, and a photoperiod simulating the natural light-dark cycle (12 h light:12 h dark).

### Appendix B.2. Supplementary Explanation on Sample Collection Procedures

Plasma was separated by centrifugation (3000 rpm, 10 min, 4 °C) and divided into three aliquots (−20 °C for biochemical analysis; −80 °C for free amino acids (FAA) and metabolite analyses). Liver tissues from three randomly selected fish per tank were excised and divided into five portions. One portion was stored at −20 °C for antioxidant capacity analysis, while the remaining four were stored at −80 °C for total m^6^A level quantification, SAM content measurement, gene expression profiling, and m^6^A methylome analysis. Dorsal muscle samples were collected post skin removal (both flanks) and stored at −80 °C for amino acid hydrolysis. Moreover, three other fish were selected from each tank for immediate liver dissection, and mitochondrial membrane potential (MMP) and reactive oxygen species (ROS) levels were measured.

### Appendix B.3 Supplementary Explanation on Sample Treatment Procedures for Hydrolyzed Amino Acids (HAA) and Free Amino Acids (FAA) Analysis

The amino acid composition and muscle content (including taurine) were quantified after acid hydrolysis. Muscle samples (0.1 g) were treated with 8 mL of 6 M HCl, flushed with N_2_ gas, and hydrolyzed at 120 °C for 22–24 h. After hydrolysis, 4.8 mL of 10 M NaOH was added for neutralization, followed by dilution to 25 mL with distilled water. The solution was filtered through double-layer filter paper, and the HAA were analysed using an amino acid analyser (Agilent 1100, USA).

High-performance liquid chromatography (HPLC) was used to measure FAA composition and content in plasma. Plasma samples were diluted with an equal volume of 10 g/100 mL trichloroacetic acid (final concentration: 5%) and filtered using double-layer filter paper. Filtrates were analyzed using a Waters 2695 HPLC system (USA). Mobile phase A consisted of 0.0225% (*v*/*v*) triethylamine and 0.8% (m/v) sodium acetate, while mobile phase B comprised a mixture of 2% (m/v) sodium acetate buffer (pH 7.2), acetonitrile, and methanol in a 1:2:2 (*v*/*v*) ratio.

### Appendix B.4. Supplementary Explanation on Plasma Metabolomics Analysis Procedures

Plasma metabolites were analyzed using LC-MS. Plasma samples (100 μL) were mixed with 400 μL of 80% methanol aqueous solution, vortexed, and centrifuged for 20 min at 15,000× *g*. A portion of the supernatant was then diluted with mass spectrometry-grade water to adjust the methanol concentration to 53%, followed by a second centrifugation at 15,000× *g* for 20 min. The final supernatant was examined for LC-MS analysis using a Vanquish UHPLC system coupled with a mass spectrometer (Q Exactive™ HF-X, Germany).

### Appendix B.5. Supplementary Explanation on Hepatic m6A Level and SAM Content Analysis Procedures

Total RNA was isolated from hepatic tissue *via* the RNAiso Plus extraction kit (TaKaRa, Dalian, China) as per the manufacturer’s protocol. The concentration and purity of the extracted RNA were evaluated using a NanoDrop ND-1000 UV-Vis spectrophotometer (NanoDrop Technologies, Wilmington, DE, USA). An RNA input of 200 ng was used to quantify N6-methyladenosine (m^6^A) modifications in hepatic RNA using the EpiQuik™ m^6^A RNA Methylation Quantification Kit (Colorimetric; Beijing Chunye Technology Co., Ltd.). The assay procedure included sequential steps of RNA immobilization (binding to assay wells), m^6^A-specific antibody capture, and signal detection based on colorimetric reading for quantitative analysis.

Liver SAM content was quantified using an ELISA-based approach. Liver tissues (0.1 g) were homogenized in 0.9 mL of sterile physiological saline (4 °C) and processed in an ice bath. The homogenate was spun at 3000 rpm for 10 min to obtain a 10% liver homogenate and quantified using a fish SAM ELISA kit (Shanghai ChaoRui Biotechnology Co., Ltd.). Tissue homogenate, standard solutions, and HRP-labeled detection antibodies were sequentially added to SAM antibody-coated wells. After incubation and washing, TMB substrate was added for chromogenic development. Absorbance was recorded at 450 nm using a microplate reader, and SAM concentrations were calculated accordingly.

### Appendix B.6. Supplementary Explanation on Hepatic Gene Expression Analysis Procedures

Gene expression levels in liver tissue were determined *via* qRT-PCR analysis. Total content of RNA was extracted using the RNAiso Plus kit (TaKaRa Biotech Co., Ltd., Dalian, China) as per the manufacturer’s protocol. The concentration and purity of extracted RNA were evaluated using a NanoDrop ND-1000 UV-Vis spectrophotometer (NanoDrop Technologies, Wilmington, DE, USA). First-strand cDNA was synthesized from the purified RNA using the PrimeScript™ RT reagent kit (TaKaRa Biotech Co., Ltd., Dalian, China). Target gene transcripts were quantified using a CFX96 Touch real-time PCR detection system (Bio-Rad, USA). Primers were designed using the NCBI Primer Blast tool (Table A2) and synthesised by Shanghai BioEngineering Co., Ltd. *β-actin* was selected as the housekeeping gene, and relative transcription levels were analyzed using the 2^−ΔΔCT^ method.

## References

[1] Serra V., Pastorelli G., Tedesco D.E.A., Turin L., Guerrini A. (2024). Alternative protein sources in aquafeed: Current scenario and future perspectives. Vet. Anim. Sci..

[2] Daniel N. (2018). A review on replacing fish meal in aqua feeds using plant protein sources. Int. J. Fish. Aquat. Stud..

[3] Terova G., Ceccotti C., Ascione C., Gasco L., Rimoldi S. (2020). Effects of partially defatted *hermetia illucens* meal in rainbow trout diet on hepatic methionine metabolism. Animals.

[4] Hussain S.M., Bano A.A., Ali S., Rizwan M., Adrees M., Zahoor A.F., Sarker P.K., Hussain M., Arsalan M.Z.-H., Yong J.W.H. (2024). Substitution of fishmeal: Highlights of potential plant protein sources for aquaculture sustainability. Heliyon.

[5] Du Y., Lin X., Shao X., Zhao J., Xu H., de Cruz C.R., Xu Q. (2024). Effects of supplementing coated methionine in a high plant-protein diet on growth, antioxidant capacity, digestive enzymes activity and expression of TOR signaling pathway associated genes in gibel carp, *Carassius auratus gibelio*. Front. Immunol..

[6] Shan L.-L., Li X.-Q., Zheng X.-M., Gan T., Guo T., Leng X.-J. (2017). Effects of feed processing and forms of dietary methionine on growth and IGF-1 expression in Jian carp. Aquac. Res..

[7] Chen Z., Liu Y., Li Y., Yang P., Hu H., Yu G., Ai Q., Xu W., Zhang W., Zhang Y. (2018). Dietary arginine supplementation mitigates the soybean meal induced enteropathy in juvenile turbot, *Scophthalmus maximus * L. Aquac. Res..

[8] Li P., Yin Y.-L., Li D., Kim S.W., Wu G. (2007). Amino acids and immune function. Br. J. Nutr..

[9] Wu G. (2009). Amino acids: Metabolism, functions, and nutrition. Amino Acids.

[10] Wang L., Gao C., Wang B., Wang C., Sagada G., Yan Y. (2023). Methionine in fish health and nutrition: Potential mechanisms, affecting factors, and future perspectives. Aquaculture.

[11] Zhou Y., He J., Su N., Masagounder K., Xu M., Chen L., Liu Q., Ye H., Sun Z., Ye C. (2021). Effects of DL-methionine and a methionine hydroxy analogue (MHA-Ca) on growth, amino acid profiles and the expression of genes related to taurine and protein synthesis in common carp (*Cyprinus carpio*). Aquaculture.

[12] Feng Y., Yang L., Zhu Y.W., Wang W.C. (2019). Methionine regulates the major physiological functions of animals. Sci. Sin. Vitae.

[13] Martínez Y., Li X., Liu G., Bin P., Yan W., Más D., Valdivié M., Hu C.-A.A., Ren W., Yin Y. (2017). The role of methionine on metabolism, oxidative stress, and diseases. Amino Acids.

[14] Pan Y., Ma P., Liu Y., Li W., Shu Y. (2018). Multiple functions of m_6_A RNA methylation in cancer. J. Hematol. Oncol..

[15] Chen X.-Y., Zhang J., Zhu J.-S. (2019). The role of m_6_A RNA methylation in human cancer. Mol. Cancer.

[16] Zhang C., Fu J., Zhou Y. (2019). A review in research progress concerning m_6_A methylation and immunoregulation. Front. Immunol..

[17] Gebeyew K., Yang C., Mi H., Cheng Y., Zhang T., Hu F., Yan Q., He Z., Tang S., Tan Z. (2022). Lipid metabolism and m_6_A RNA methylation are altered in lambs supplemented rumen-protected methionine and lysine in a low-protein diet. J. Anim. Sci. Biotechnol..

[18] Li S., Wang Y., Xu A., Zhao B., Xia Y., He Y., Xue H., Li S. (2024). Dietary selenomethionine reduced oxidative stress by resisting METTL3-mediated m_6_A methylation level of Nrf2 to ameliorate LPS-induced liver necroptosis in laying hens. J. Nutr. Biochem..

[19] Miao L.-H., Lin Y., Pan W.-J., Huang X., Ge X.-P., Ren M.-C., Zhou Q.-L., Liu B. (2017). Identification of differentially expressed microRNAs associate with glucose metabolism in different organs of blunt snout bream (*Megalobrama amblycephala*). Int. J. Mol. Sci..

[20] Liao Y.J., Ren M.C., Liu B., Sun S.M., Cui H.H., Xie J., Zhou Q.L., Pan L.K., Chen R.L., Ge X.P. (2014). Dietary methionine requirement of juvenile blunt snout bream (*Megalobrama amblycephala*) at a constant dietary cystine level. Aquac. Nutr..

[21] Liang H.-L., Ren M.-C., Habte-Tsion H.-M., Mi H.-F., Ge X.-P., Xie J., Xi B.-W., Zhou Q.-L., Miao L.-H. (2016). Dietary methionine requirement of pre-adult blunt snout bream, (*Megalobrama amblycephala* Yih, 1955). J. Appl. Ichthyol..

[22] Fan C., Ma Y., Chen S., Zhou Q., Jiang H., Zhang J., Wu F. (2021). Comprehensive Analysis of the Transcriptome-Wide m_6_A Methylation Modification Difference in Liver Fibrosis Mice by High-Throughput m_6_A Sequencing. Front. Cell Dev. Biol..

[23] Jiang W., Lin Y., Qian L., Lu S., Shen H., Ge X., Miao L. (2024). Mulberry Leaf Polysaccharides Attenuate Oxidative Stress Injury in Peripheral Blood Leukocytes by Regulating Endoplasmic Reticulum Stress. Antioxidants.

[24] Ji K., Liang H., Ren M., Ge X., Pan L., Yu H. (2021). Nutrient metabolism in the liver and muscle of juvenile blunt snout bream (*Megalobrama amblycephala*) in response to dietary methionine levels. Sci. Rep..

[25] Luo Z., Liu Y., Mai K., Tian L., Yang H., Tan X., Liu D. (2005). Dietary L-methionine requirement of juvenile grouper *Epinephelus coioides* at a constant dietary cystine level. Aquaculture.

[26] Fang C.-C., Feng L., Jiang W.-D., Wu P., Liu Y., Kuang S.-Y., Tang L., Liu X.-A., Zhou X.-Q. (2021). Effects of dietary methionine on growth performance, muscle nutritive deposition, muscle fibre growth and type I collagen synthesis of on-growing grass carp (*Ctenopharyngodon idella*). Br. J. Nutr..

[27] Zhou F., Xiao J.X., Hua Y., Ngandzali B.O., Shao Q.J. (2011). Dietary l-methionine requirement of juvenile black sea bream (*Sparus macrocephalus*) at a constant dietary cystine level. Aquac. Nutr..

[28] Hu Y., Cai M., Zhong H., Chu W., Hu Y. (2021). A study on how methionine restriction decreases the body’s hepatic and lipid deposition in rice field Eel (*Monopterus albus*). Int. J. Mol. Sci..

[29] Lu Y., Wu Z., Song Z., Xiao P., Liu Y., Zhang P., You F. (2016). Insight into the heat resistance of fish via blood: Effects of heat stress on metabolism, oxidative stress and antioxidant response of olive flounder *Paralichthys olivaceus* and turbot *Scophthalmus maximus*. Fish Shellfish Immunol..

[30] Hernansanz-Agustín P., Enríquez J.A. (2021). Generation of reactive oxygen species by mitochondria. Antioxidants.

[31] Correia-Álvarez E., Keating J.E., Glish G., Tarran R., Sassano M.F. (2020). Reactive oxygen species, mitochondrial membrane potential, and cellular membrane potential are predictors of e-liquid induced cellular toxicity. Nicotine Tob. Res..

[32] Li X., Zhao Y., Yin J., Lin W. (2020). Organic fluorescent probes for detecting mitochondrial membrane potential. Coord. Chem. Rev..

[33] Zhang X., Zhao Z., Wang X., Zhang S., Zhao Z., Feng W., Xu L., Nie J., Li H., Liu J. (2024). Deprivation of methionine inhibits osteosarcoma growth and metastasis via C1orf112-mediated regulation of mitochondrial functions. Cell Death Dis..

[34] Li J., Xu W., Lai W., Kong A., Zhang Z., Pang Y., Wang Z., Shentu J., Wu X., Mai K. (2021). Effect of dietary methionine on growth performance, lipid metabolism and antioxidant capacity of large yellow croaker (*Larimichthys crocea*) fed with high lipid diets. Aquaculture.

[35] Gomez J., Caro P., Sanchez I., Naudi A., Jove M., Portero-Otin M., Lopez-Torres M., Pamplona R., Barja G. (2009). Effect of methionine dietary supplementation on mitochondrial oxygen radical generation and oxidative DNA damage in rat liver and heart. J. Bioenerg. Biomembr..

[36] Song Y.-F., Gao Y., Hogstrand C., Li D.-D., Pan Y.-X., Luo Z. (2018). Upstream Regulators of Apoptosis Mediates Methionine-Induced Changes of Lipid Metabolism. Cellular Signalling.

[37] Yun Y., Song D., He Z., Mi J., Wang L., Nie G. (2022). Effects of methionine supplementation in plant protein based diet on growth performance and fillet quality of juveniles Yellow River carp (*Cyprinus carpio haematopterus*). Aquaculture.

[38] Teodósio R., Engrola S., Cabano M., Colen R., Masagounder K., Aragão C. (2022). Metabolic and nutritional responses of Nile tilapia juveniles to dietary methionine sources. Br. J. Nutr..

[39] Liu Y., Qian R., Xu Q., Zhao J. (2025). Integrative Metabolomic and Transcriptomic Analyses Reveal the Impact of Methionine Supplementation to Gibel Carp (*Carassius auratus gibelio*). Fishes.

[40] Sim W.-C., Han I., Lee W., Choi Y.-J., Lee K.-Y., Kim D.G., Jung S.-H., Oh S.-H., Lee B.-H. (2016). Inhibition of homocysteine-induced endoplasmic reticulum stress and endothelial cell damage by l-serine and glycine. Toxicol. Vitr..

[41] Michelato M., Furuya W.M., Gatlin D.M. (2018). Metabolic responses of Nile tilapia Oreochromis niloticus to methionine and taurine supplementation. Aquaculture.

[42] Nwanna L.C., Lemme A., Metwally A., Schwarz F.J. (2012). Response of common carp (*Cyprinus carpio* L.) to supplemental DL-methionine and different feeding strategies. Aquaculture.

[43] Choo P.-S., Smith T.K., Cho C.Y., Ferguson H.W. (1991). Dietary excesses of leucine influence growth and body composition of rainbow trout. J. Nutr..

[44] Coloso R.M., Murillo-Gurrea D.P., Borlongan I.G., Catacutan M.R. (1999). Sulphur amino acid requirement of juvenile Asian sea bass Lates calcarifer. J. Appl. Ichthyol..

[45] Somani S.T., Zeigler M., Fay E.E., Leahy M., Bermudez B., Totah R.A., Hebert M.F. (2021). Changes in erythrocyte membrane epoxyeicosatrienoic, dihydroxyeicosatrienoic, and hydroxyeicosatetraenoic acids during pregnancy. Life Sci..

[46] Hidayat R., El-Ghiaty M.A., Shoieb S.M., Alqahtani M.A., El-Kadi A.O. (2023). The effects of 16-HETE enantiomers on hypertrophic markers in human fetal ventricular cardiomyocytes, RL-14 cells. Eur. J. Drug Metab. Pharmacokinet..

[47] Krzystek-Korpacka M., Fleszar M.G., Fortuna P., Gostomska-Pampuch K., Lewandowski Ł., Piasecki T., Kosyk B., Szeląg A., Trocha M. (2021). Modulation of prostanoids profile and counter-regulation of SDF-1α/CXCR4 and VIP/VPAC2 expression by sitagliptin in non-diabetic rat model of hepatic ischemia-reperfusion injury. Int. J. Mol. Sci..

[48] Sherlock M., Behan L.A., Hannon M.J., Alonso A.A., Thompson C.J., Murray R.D., Crabtree N., Hughes B.A., Arlt W., Agha A. (2015). The modulation of corticosteroid metabolism by hydrocortisone therapy in patients with hypopituitarism increases tissue glucocorticoid exposure. Eur. J. Endocrinol..

[49] Gastaldello A., Livingstone D.E., Abernethie A.J., Tsang N., Walker B.R., Hadoke P.W., Andrew R. (2017). Safer topical treatment for inflammation using 5α-tetrahydrocorticosterone in mouse models. Biochem. Pharmacol..

[50] Sun Y., Wang X., Zhou Y., Zhang J., Cui W., Wang E., Du J., Wei B., Xu X. (2021). Protective effect of metformin on BPA-induced liver toxicity in rats through upregulation of cystathionine β synthase and cystathionine γ lyase expression. Sci. Total Environ..

[51] Zhao S., Zhong Y., Fu X., Wang Y., Ye P., Cai J., Liu Y., Sun J., Mei Z., Jiang Y. (2019). H3K4 methylation regulates LPS-induced proinflammatory cytokine expression and release in macrophages. Shock.

[52] Kakisaka K., Cazanave S.C., Fingas C.D., Guicciardi M.E., Bronk S.F., Werneburg N.W., Mott J.L., Gores G.J. (2012). Mechanisms of lysophosphatidylcholine-induced hepatocyte lipoapoptosis. Am. J. Physiol.-Gastrointest. Liver Physiol..

[53] Kahl M., Xu Z., Arumugam S., Edens B.M., Fischietti M., Zhu A.C., Platanias L.C., He C., Zhuang X., Ma Y.C. (2024). m_6_A RNA methylation regulates mitochondrial function. Hum. Mol. Genet..

[54] Li T., Tan Y.-T., Chen Y.-X., Zheng X.-J., Wang W., Liao K., Mo H.-Y., Lin J., Yang W., Piao H.-L. (2023). Methionine deficiency facilitates antitumour immunity by altering m_6_A methylation of immune checkpoint transcripts. Gut.

[55] Xing Z., Tu B.P. (2025). Mechanisms and rationales of SAM homeostasis. Trends Biochem. Sci..

[56] Kwasek K., Terova G., Lee B.-J., Bossi E., Saroglia M., Dabrowski K. (2014). Dietary methionine supplementation alters the expression of genes involved in methionine metabolism in salmonids. Aquaculture.

[57] Ceccotti C., Biasato I., Gasco L., Caimi C., Bellezza Oddon S., Rimoldi S., Brambilla F., Terova G. (2022). How different dietary methionine sources could modulate the hepatic metabolism in rainbow trout?. Curr. Issues Mol. Biol..

[58] Wang J., Zhang J., Ma Y., Zeng Y., Lu C., Yang F., Jiang N., Zhang X., Wang Y., Xu Y. (2021). WTAP promotes myocardial ischemia/reperfusion injury by increasing endoplasmic reticulum stress via regulating m_6_A modification of ATF4 mRNA. Aging.

[59] Wang P.-X., Zhu L., Xiang M., Zhang R., Zheng X., Zheng Z., Li K. (2025). FTO Alleviates Hepatic Ischemia-Reperfusion Injury by Regulating Apoptosis and Autophagy. Gastroenterol. Res. Pract..

[60] Jin C., Li Y., Su Y., Guo Z., Wang X., Wang S., Zhang F., Zhang Z., Shao J., Zheng S. (2020). Novel copper complex CTB regulates methionine cycle induced TERT hypomethylation to promote HCC cells senescence via mitochondrial SLC25A26. Cell Death Dis..

[61] Chen L., Liu Y., Zhang J., Song T., Wu J., Ren Z. (2025). AMPK regulates ARF1 localization to membrane contact sites to facilitate fatty acid transfer between lipid droplets and mitochondria. Cell Death Dis..

[62] Farhan M., Silva M., Li S., Yan F., Fang J., Peng T., Hu J., Tsao M.-S., Little P., Zheng W. (2020). The role of FOXOs and autophagy in cancer and metastasis—Implications in therapeutic development. Med. Res. Rev..

[63] Javed R., Mari M., Trosdal E., Duque T., Paddar M.A., Allers L., Mudd M.H., Claude-Taupin A., Akepati P.R., Hendrix E. (2025). ATG9A facilitates the closure of mammalian autophagosomes. J. Cell Biol..

[64] Liu M., Chen Y., Fan X., Gu J., Xing S. (2025). Beclin1-mediated vascular autophagy negatively regulates angiogenesis and secondary neural damage in the thalamus following cerebral cortical infarction. IBRO Neurosci. Rep..

[65] Gupta S., Afzal M., Agrawal N., Almalki W.H., Rana M., Gangola S., Chinni S.V., Kumar K.B., Ali H., Singh S.K. (2025). Harnessing the FOXO-SIRT1 axis: Insights into cellular stress, metabolism, and aging. Biogerontology.

[66] Kim J.Y., Mondaca-Ruff D., Singh S., Wang Y. (2022). SIRT1 and autophagy: Implications in endocrine disorders. Front. Endocrinol..

[67] Li X., Zheng S., Ma X., Cheng K., Wu G. (2021). Use of alternative protein sources for fishmeal replacement in the diet of largemouth bass (*Micropterus salmoides*). Part I: Effects of poultry by-product meal and soybean meal on growth, feed utilization, and health. Amino Acids.

[68] Wang M., Chen X., Li S., Wang L., Tang H., Pu Y., Zhang D., Fang B., Bai X. (2024). A crosstalk between autophagy and apoptosis in intracerebral hemorrhage. Front. Cell. Neurosci..

[69] Machado M., Serra C.R., Oliva-Teles A., Costas B. (2021). Methionine and tryptophan play different modulatory roles in the European seabass (Dicentrarchus labrax) innate immune response and apoptosis signaling—An in vitro study. Front. Immunol..

[70] Yao Q., Ke Z., Guo S., Yang X., Zhang F., Liu X., Chen X., Chen H., Ke H., Liu C. (2018). Curcumin protects against diabetic cardiomyopathy by promoting autophagy and alleviating apoptosis. J. Mol. Cell. Cardiol..

